# A Site-Specific MiniAp4–Trastuzumab Conjugate Prevents Brain Metastasis

**DOI:** 10.1021/acs.molpharmaceut.4c01091

**Published:** 2025-02-10

**Authors:** Mariam Masmudi-Martín, Benjamí Oller-Salvia, María Perea, Meritxell Teixidó, Manuel Valiente, Ernest Giralt, Macarena Sánchez-Navarro

**Affiliations:** a Brain Metastasis Group, CNIO, Madrid 28029, Spain; b Institute for Research in Biomedicine (IRB Barcelona), Barcelona Institute of Science and Technology (BIST), Barcelona 08028, Spain; c Department of Inorganic and Organic Chemistry, University of Barcelona, Barcelona 08028, Spain; d Grup d’Enginyeria de Materials, Institut Químic de Sarrià (IQS), Universitat Ramon Llull, Barcelona 08017, Spain; e Department of Biochemistry and Molecular Pharmacology, Instituto de Parasitologia y Biomedicina “López-Neyra” (CSIC), Granada 18100, Spain

**Keywords:** brain shuttle peptide, trastuzumab, brain metastasis

## Abstract

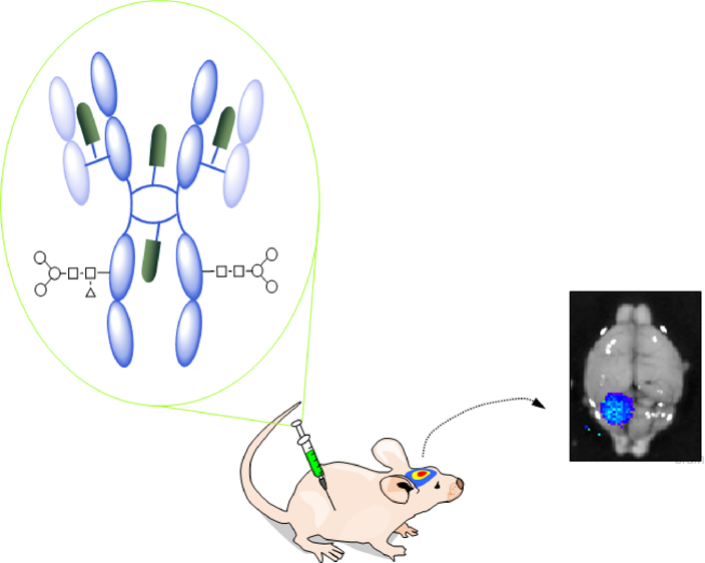

Monoclonal antibodies (mAbs) are changing cancer treatments. However, the presence of the blood–brain barrier (BBB) and the blood–tumor barrier (BTB) limits the use of mAbs to treat brain cancer or brain metastasis. Molecules that hijack endogenous transport mechanisms on the brain endothelium (brain shuttles) have been shown to increase the transport of large molecules and nanoparticles across the BBB. Among these shuttles, protease-resistant peptides such as MiniAp-4 are particularly efficient. Here, we report the synthesis, characterization, and evaluation of site-specific mAb–brainshuttle antibody conjugates (ASC) based on the anti-HER2 mAb trastuzumab (Tz) and four molecules of MiniAp-4. The ASCs preserve the binding and cell cycle arrest capacity of Tz. MiniAp-4 ASC displays enhanced transport across an *in vitro* BBB cellular model with respect to Tz and Tz conjugated to Angiopep-2, the brain shuttle that has advanced the most in clinical trials. More importantly, evaluation of Tz-MiniAp4 in a murine brain metastasis model demonstrated that the protease-resistant peptide showed preferential transport across the BBB/BTB, displaying a marked therapeutic effect and protecting against metastasis development. The technology described herein could be applied to any antibody of interest to treat central nervous system-related diseases. MiniAp-4 enhances the brain transport of the monoclonal antibody trastuzumab, preventing brain metastasis.

## Introduction

Monoclonal antibodies (mAbs) have revolutionized the treatment of several diseases, in particular cancer. Antibody-based treatments target leukemia and solid tumors in many organs. However, brain tumors remain practically intractable with biotherapeutics and most small molecules. One of the main challenges in treating brain tumors is overcoming the blood–brain barrier (BBB) and the blood–tumor barrier (BTB) in therapeutic amounts,^1,2^ since only 0.1–0.2% of peripherally injected doses of mAbs reach the brain parenchyma.^3,4^ The BBB consists of specialized endothelial cells, which are tightly connected and surrounded by astrocyte end-feet and pericytes and ensure brain isolation.^5−8^ Although the BBB is replaced by the leaky BTB, it may be intact at the tumor margins and in small brain metastases, hampering drug access.

Brain metastasis (BM) is a major complication in several types of cancers, particularly in lung, melanoma, and breast cancer.^9−11^ Breast cancer BM is especially prevalent, affecting 24% of women with stage IV breast cancer.^12^ Breast tumors overexpressing human epidermal growth factor receptor (HER2) and triple-negative breast tumors show a higher incidence of BM,^13,14^ which is the major contribution to reduced survival.^15^ The application of mAbs or antibody–drug conjugates (ADCs) has proven highly efficacious in these types of cancer with limited efficacy in the treatment of BM.^16^ Anti-Her2 antibody–drug conjugates based on trastuzumab (Tz) (herceptin), an FDA-approved antibody against HER2, have been evaluated in clinical settings with partially positive results. Treatment of Her2-positive metastatic breast cancer with trastuzumab-emtansine (NCT01702571) resulted in *a* ≥ 30% reduction of the sum of the diameters of the BMs in almost 50% of BM-positive patients.^17^ Trastuzumab-deruxtecan (NCT03248492) therapy led to a median duration of progression-free survival of 16.4 months, indicating no differences between patients with and without previously developed BM.^18^ However, more trials are needed to be able to compare with the results obtained with small molecules, such as tucatinib, a brain-penetrant tyrosine kinase inhibitor.^19^ Indeed, current clinical trials are evaluating the effect of trastuzumab-emtansine (NCT03975647) or trastuzumab-deruxtecan (NCT04539938) in combination with tucatinib^19^ to prevent HER2+ breast cancer BM. Despite the encouraging results from antibody–drug conjugates, the lack of antibody penetration across the BBB and BTB is still a major issue.^16^

Several approaches have been explored to increase the brain penetration capacity of antibodies. In this regard, methods like direct injection and temporal disruption of the BBB may entail high risks for the patient.^20^ Consequently, considerable efforts have been devoted to the development of ligands that hijack endogenous transport mechanisms across the brain endothelium. These ligands, dubbed brain shuttles, include antibody derivatives,^21−23^ endogenous proteins,^24^ peptides,^25−200^ and small molecules. In this context, peptides stand out because they can combine the high selectivity of biologics and the chemical accessibility, small size, and low immunogenicity of small molecules. Furthermore, the lower affinity of peptides for their targets than that shown by antibodies may be beneficial for brain delivery as it may enable bypassing the endolysosomal pathway.^22,25,28,29^

Our laboratories have worked extensively on the generation of BBB-shuttle peptides from different sources, ranging from venoms to chemical and phage display libraries.^30−35^ One of our main contributions is the demonstration that high resistance to serum proteases is relevant to producing efficacious BBB shuttles. One of the BBB shuttles with most potential is MiniAp-4.^31^ Derived from bee venom, this cyclic peptidomimetic shows a high resistance to proteolysis and negligible toxicity and immunogenicity. In mice, MiniAp-4 was demonstrated to cross the BBB and reach the brain parenchyma. Given that this brain shuttle was shown to increase the BBB transport of a wide variety of cargos, including model proteins and nanoparticles, we hypothesized that it might also increase the transport of large and therapeutically relevant proteins such as antibodies.

In this work, we have addressed the long-standing challenge of increasing antibody transport across the BBB for the treatment of BM. To this end, we conjugated MiniAp-4 to Tz, which is widely used to treat breast cancer.^36,37^ Although some reports describe a limited degree of brain penetration for Tz,^38,39^ its low efficacy in the treatment of HER2+ BM is most likely related to the poor brain penetration. An antibody–brain shuttle conjugate (ASC) of Tz and Angiopep-2, a brain shuttle that has reached advanced stages of clinical development, has been reported previously.^40,41^ However, the limited efficacy of the conjugated shuttle could be due to the low protease resistance of the peptide and the heterogeneous mixture obtained by randomly conjugating the peptide to surface lysine residues.

The generation of homogeneous constructs by site-specific modification of the antibody in antibody–drug conjugates, a class of cancer therapeutics that has surged in the past decade, has proven key to improving pharmacokinetics and binding, thereby enhancing their therapeutic index. Recently, a homogeneous ASC of angiopep-2, prepared by enzymatically incorporating reactive handles on an anti-EGFR2 antibody, has been shown to have improved transport in a BBB model.^42^

Here, we prepared a homogeneous ASC of Tz with four copies of MiniAp-4 (Tz-MiniAp4) or the control peptide Angiopep-2 (Tz-Ang2). The conjugation was achieved by reducing interchain disulfide bridges and rebridging them using dibromomaleimide (DBM), which allows high control over the number of peptides anchored to each antibody molecule. The new constructs were fully characterized by gel electrophoresis (SDS-PAGE) and mass spectrometry (MS), and their binding and functional properties were evaluated *in vitro*. In addition, a human BBB cell-based model was used to determine the capacity of these constructs to penetrate the brain. Although both ASCs crossed the endothelial cell monolayer, Tz-MiniAp4 showed a significantly greater penetration than Tz-Ang2. Following the experiments on cells, we studied the conjugates *in vivo*. Accumulation of Tz in mouse brain parenchyma was 60% higher for Tz-MiniAp4 and the brain-to-plasma ratio 40% higher than the reference conjugate. Finally, after these promising results, Tz-MiniAp4 was studied in a mouse model of HER2+ BM and was found to be protective against BM.

## Experimental Section

### Materials

Protected amino acids were supplied by Iris Biotech (Marktredwitz, Germany). ChemMatrix resin was purchased from PCAS BioMatrix (QC, Canada). Diisopropylethylamine (DIEA), *N*,*N*′-diisopropylcarbodiimide (DIC), and ninhydrin were supplied by Fluka Chemika (Buchs, Switzerland). Solvents for peptide synthesis and liquid chromatography were provided by SDS (Barcelona, Spain). Trifluoroacetic acid (TFA) was purchased from Scharlau (Barcelona, Spain). The other chemicals used were obtained from Aldrich (Milwaukee, Wisconsin) and were of the highest purity commercially available. All compounds are >95% pure by HPLC or UPLC analysis.

Cell culture-treated plates and flasks were purchased from Corning Costar. Culture medium was acquired from Lonza. The XTT cell proliferation kit was purchased from Biological Industries (Cromwell, Connecticut). Pierce iodination beads were obtained from Pierce. Desalting columns (MiniTrap and MidiTrap G-25) were obtained from GE Healthcare.

#### Synthesis of (3,4-Dibromo-2,5-dioxo-2,5-dihydro-1*H*-pyrrol-1-yl)acetic Acid

3,4-Dibromo-2,5-dioxo-2,5-dihydro-1*H*-pyrrol-1-yl)acetic acid was prepared as described in Behrens et al.^43^ In brief, glycine (0.294 mg, 3.91 mmol) was added to a solution of 3,4-dibromofuran-2,5-dione (1 g, 3.91 mmol) in acetic acid (20 mL), and the solution was stirred at room temperature for 10 min until all of the solids dissolved. The reaction mixture was heated to 100 °C overnight. The solution was concentrated under a vacuum and purified by silica gel chromatography (eluent DCM/MeOH 9:1). The concentration of pure fractions afforded 1.08 g (3.4 mmol, 89% yield) of the DBM derivative 2-(3,4-dibromo-2,5-dioxo-2,5-dihydro-1H-pyrrol-1-yl)acetic acid.

^1^H NMR (400 MHz, CD_3_OD): δ 4.32 (s, 2H) ppm.

^13^C NMR (101 MHz, CD_3_OD): δ 170, 164, 129, 40 ppm.

*m*/*z*: 309.81, 311.84, 313.87 [M- H]^−^.

#### Peptide Synthesis and Chromatography

Angiopep-2 (DBM-TTDS-TFFYGGSRGKRNNFKTEEY-OH) and MiniAp4 (DBM-TTDS-[Dap](&)KAPETALD(&)-NH_2_) were prepared manually using Fmoc/tBu solid-phase synthesis. They were prepared on a 250 μmol scale on Rink Amide ChemMatrix resin. Between the coupling and deprotection steps, three washes with 5 mL of DMF for 30 s were performed.

#### Initial Conditioning of the Resin

ChemMatrix resin with a substitution of 0.49 mmol/g was conditioned by washing with MeOH (5 × 30 s), DMF (5 × 30 s), DCM (5 × 30 s), and 1% trifluoroacetic acid (TFA) in DCM (2 × 10 min), followed by DCM (5 × 30 s), DMF (5 × 30 s), and DCM (5 × 30 s). It was then washed with 5% DIEA in DCM (2 × 30 s) and finally with DCM (5 × 30 s) and DMF (5 × 30 s).

#### Identification Test

The Kaiser^44^ colorimetric test was used to detect primary amines bound to the resin.

#### Fmoc Group Removal

The Fmoc group was removed by treating the resin with 20% (v/v) piperidine in DMF. The resin was then washed with DMF (3 × 30 s).

#### Coupling Methods

Coupling reactions were performed using 3 eq. Fmoc-(Amino Acid)–OH, 3 eq. Oxyma, and 3 eq. DIC all in 1:1 DMF:DCM for 30–45 min. Washing with DMF/DCM was followed by a ninhydrin test to check for successful coupling. Next, 20% piperidine in DMF was added (1 × 1, 2 × 10 min), followed by washing and another ninhydrin test. This cycle was repeated until all the amino acids had been added, including the dibromomaleimide moiety (3 equiv of all coupling reagents).

#### Cleavage and Deprotection of Side Chains

After completion of the peptide chain, the resin was washed with DCM (5 × 30 s) and dried by suction for 15 min. The peptides were cleaved from the resin with the concomitant removal of the side-chain protecting groups using the following cleavage cocktail: TFA, H_2_O, and triisopropylsilane (TIS) (95:2.5:2.5). After 2 h of cleavage, the solvent was evaporated by applying a stream of N_2_. The residue was washed three times by suspension in cold *tert*-butyl methyl ether and subsequent centrifugation. Finally, the cleaved peptides were dissolved in H_2_O/MeCN (1:1) and lyophilized.

#### Peptide Purification

Peptides were purified on a Teledyne Isco flash system using a 30 g prepacked RediSep C18 column with a H_2_O (0.1% TFA)–MeCN (0.1% TFA) gradient. Fractions were collected by monitoring their absorbance at 254 nm. The fractions were evaluated by LC-ESI-MS. The fractions with enough purity were collected together and freeze-dried. The identity of the peptides synthesized was confirmed by UPLC-MS, while their purity was assessed by UPLC.

#### UPLC Analysis

UPLC chromatograms were obtained on an Acquity high-class system (PDA detector, sample manager FNT, and Quaternary solvent manager) using an Acquity BEH C18 (50 × 2 mm × 1.7 μm) column. The flow rate was 0.61 mL/min, and MeCN (0.036% TFA) and H_2_O (0.045% TFA) were used as solvents. In all cases, 2 min linear gradients were used.

#### UPLC-MS Analysis

Chromatograms and spectra were recorded on a Waters high-class system (PDA detector, sample manager FNT, and quaternary solvent manager) coupled to an electrospray ion source ESI-MS Micromass ZQ using MassLynx 4.1 software (Waters, Milford, Massachusetts). Using a BEH C_18_ column (50 × 2.1 mm × 1.7 μm, Waters). The flow rate was 0.6 mL/min, and MeCN (0.07% formic acid) and H_2_O (0.1% formic acid) were used as solvents. Samples were analyzed with positive ionization: the ion spay voltage was 30 V, and the capillary temperature was 1 kV.

#### Amino Acid Analysis

Amino acid analysis was performed to assess the amino acids present and the amounts obtained for each peptide. To this end, ion exchange chromatographic analysis after acid hydrolysis was performed. The samples were hydrolyzed with 6 M HCl at 110 °C for 16 h. They were then evaporated to dryness at reduced pressure and dissolved in 20 mM aqueous HCl. Finally, the amino acids were modified using the AccQ-Tag protocol from Waters and analyzed by ion exchange HPLC.

For amino acid analysis, 100 μL of peptide (1 mg/mL) was added to 100 μL of HCl (12 M) and 20 μL of aminoquinolyl-*N*-hydroxysuccinimidyl carbamate derivatization reagent. This mixture was left overnight at 110 °C. The liquid was fully evaporated, and 200 μL of 20 mM HCl was added before the Waters AccQ-Tag protocol was performed.

#### Trastuzumab Modification

All protein experiments were performed in microcentrifuge tubes (1.5, 2, or 5 mL) at rt with mixing. All buffer solutions were prepared with Milli-Q water. Borate buffered saline (BBS) stands for 50 mM sodium borate, 50 mM NaCl, and 5 mM ethylenediaminetetraacetic acid (EDTA) at pH 8.5. Phosphate-buffered saline (PBS) stands for 10 mM sodium phosphate, 137 mM sodium chloride, and 2.7 mM potassium chloride at pH 7.4.

Tris(2-carboxyethyl)phosphine hydrochloride (TCEP) solutions of 10 mM (2.87 mgmL^–1^) were prepared in BBS immediately before use.

The concentration was measured out in Amicon Ultra-15 low binding cellulose filters with 10 kDa MWCO. Centrifugation was carried out on a Beckman Coulter Allegra 21K centrifuge operating at 3500 rcf at 4 °C.

Trastuzumab was obtained in its clinical form (Roche, lyophilized), suspended in 7.2 mL of sterile water, and the buffer exchanged completely for BBS pH 8.5 with PD10 G-25 columns (GE Healthcare). The concentration was determined by UV/vis absorbance (using ε280 = 215,380 M^–1^ cm^–1^ for trastuzumab mAb), and the protein was stored in flash frozen aliquots at −20 °C. For experiments, aliquots were thawed and used immediately. The ADC concentration was determined using the same extinction coefficient for MiniAp-4 since DBM-TTDS-MiniAp4 were found to have negligible absorbance at 280 nm and ε280 = 227,300 M^–1^ cm^–1^ for DBM-TTDS-Ang2 since each molecule of Ang2 is estimated to contribute with ε280 = 2980 M^–1^ cm^–1^ (by the Expasy ParamTool online tool).^45^

The following acronyms are used to describe antibody fragments based on their constituent heavy and light chains: heavy–heavy–light (HHL), heavy–heavy (HH), heavy–light (HL), heavy chain (HC), and light chain (LC).

Expected mass was calculated according to MS data observed for trastuzumab subunits and full antibody (LC: 23,440 kDa; HC: 50,584 kDa; HL: 74,024 kDa; HHLL: 148,048 kDa).

#### Conjugation of DBM Peptide Trastuzumab at pH 8.5

The conjugation protocol was adapted from Morais et al.^46^ In brief, trastuzumab (111 μM, 4.9 mL, 544 nmol) was diluted with BBS (pH 8.5) to a final concentration of 22.9 μM. A fresh solution of TCEP was added (10 mM, 332.2 μL, 3.26 μmol, 6 equiv), and the reaction was incubated at 37 °C for 2 h under mild agitation. TCEP was removed by SEC using PD10 G-25 columns with BBS as buffer, following the manufacturer′s instructions. Next, the DBM peptide in dry DMF (10 mM, 247 μL, 4.35 μmol, 8 equiv) was added to the reduced trastuzumab and the reaction was left at rt for 30 min. Afterward, excess reagents were removed by SEC using PD10 G-25 columns with PBS. The final conjugates were characterized by LC-MS.

#### mAb MS Analysis—Description

The LC-MS system setup was as follows. 8 μL of sample was injected automatically to a BioSuite Phenyl 1000 (Waters, 10 μm RPC 2.0 × 75 mm) column at a flow rate of 100 μL/min using an Acquity UPLC system (Waters Corporation) provided with a binary solvent manager and an automatic autosampler. Intact protein was eluted using a linear gradient from 5 to 80% B in 60 min (A = 0.1% formic acid (FA) in water, B = 0.1% FA in CH_3_CN). The column outlet was directly introduced into the electrospray ionization (ESI) source of a Waters LCT-Premier XE mass spectrometer (TOF). Capillary voltage and cone voltage were set to 3000 and 100 V, respectively. Desolvation and source temperatures were set to 350 and 120 °C, respectively. Cone and desolvation gas flow were set to 50 and 600 L/h, respectively.

The mass spectrometer acquired full MS scans (400–4000 *m*/*z*) working in the positive polarity mode.

#### Data Processing

Data were acquired with MassLynx software V4.1.SCN704 (Waters Inc.). MS spectra corresponding to the chromatographic peak were summed. Charged protein species in the resulting spectrum were deconvoluted to their zero charged average masses by using the integrated MaxEnt1 (maximum entropy) algorithm.

Output parameters were as follows: mass range 5000–70,000 and resolution 1 Da/channel. A uniform Gaussian model was used with the corresponding peak widths at half height.

#### Binding of Herceptin and Ang2 and MiniAp4 Conjugates

In vitro binding of Herceptin, Ang2, and MiniAp-4 conjugates to HER2-positive BT-474 and SK-BR-3 breast cancer cells was determined by flow cytometry. Confluent cells were detached from flasks with trypsin, which was neutralized with FBS-supplemented DMEM. Cells in suspension were washed in ice-cold PBS, counted, and separated into individual 1.5 mL tubes (10^6^ cells per tube). Binding of Herceptin and Ang2 and MiniAp-4 conjugates was performed with increasing concentrations in ice-cold PBS for 30 min at 4 °C. Cells were then washed and incubated with an anti-human-DyLight 650 secondary antibody (Abcam plc) in ice-cold PBS for 30 min at 4 °C. Cells were washed with ice-cold PBS and analyzed by flow cytometry (10,000 gated events per condition).

#### Cell Cycle Arrest

HER2-positive BT-474 and SK-BR-3 breast cancer cells and HER2-negative breast cancer cells MD-MB-231 were grown in 12-well plates in the monolayer up to 50% confluence and serum-starved overnight. Then, the cells were treated with Tz, Tz-Ang2, and Tz-MiniAp4 (100 nM) in complete media. 24 h after stimulation, the cells were trypsinized, washed twice with ice-cold PBS, fixed in 70% ethanol at 20 °C for 15 min, resuspended in RNaseA 1 mg/mL (EURx Ltd., Gdansk, Poland), and stained with propidium iodide (2.5 μg/mL). The cell cycle was analyzed with a BD LSR II flow cytometer (BD Biosciences).

#### ^125^I Protein Labeling and Quantification

Pierce Iodination Beads (Life Technologies) were used to radiolabel the mAb and its conjugates. Briefly, two beads per protein were washed with 500 μL of reaction buffer (50 mM NaPi, pH 6.5) and dried on a filter paper. In a glass vial, the beads were added with the calculated amount of carrier-free Na^125^I (1 mCi/mg protein) in 200 μL of reaction buffer. The reaction mixture was incubated for 5 min. The proteins were then added, and the reaction was carried out for 15 min with occasional mixing. The reaction was stopped by removing the solution from the reaction vessel and adding it to a PD MiniTrap G-25 column (GE Healthcare) previously equilibrated with PBS. The iodinated protein was dialyzed (Slide-A-Lyzer mini-dialysis devices, 20 kDa, 0.5 mL) overnight against PBS to further remove the unincorporated ^125^I. The radioactivity of 10 μL fractions was measured for 2 min using a Packard Cobra II Gamma Counter, and the protein concentration was determined using BCA (Thermo Scientific). The samples were diluted with Ringer Hepes to a final concentration of 100 nM.

#### Permeability Assays in the In Vitro Human BBB Cellular Model

These experiments were performed using the model developed in Prof. R. Cecchelli’s laboratory.^47^ In brief, endothelial cells derived from pluripotent stem cells and bovine pericytes were defrosted in gelatin-coated Petri dishes (Corning). Pericytes were cultured in DMEM pH 6.8 while endothelial cells were cultured in supplemented endothelial cell growth medium (sECM) (ScienCell). After 48 h, endothelial cells were seeded in 12-well Transwell inserts (8000 cell/well) and pericytes were plated in 12-well plates (50,000 cells/well) previously coated with Matrigel and gelatin, respectively. sECM medium was used for both cell lines and changed every 2–3 days. The assays were performed 7–8 days after seeding by placing inserts containing the endothelial cells into new wells without pericytes. In all experiments, Lucifer Yellow (25 μM) was added as a control of barrier integrity (Papp <15 × 10^–6^ cm/s).

To perform the transport assay (Figure 3a), 500 μL of ^125^I-labeled versions of Tz, Tz-Ang2, and Tz-MiniAp4 (100 nM) in Ringer HEPES was added to the donor compartment and 1500 μL of Ringer HEPES was introduced into the acceptor compartment. The plates were incubated for 2 h at 37 °C, and the solutions from both compartments were recovered and analyzed.

The samples were evaluated in triplicates. The amount of protein was quantified using a gamma counter and the apparent permeability using the following formula:
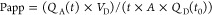
where Papp is obtained in cm/s, *Q*_A_(*t*) is the amount of compound at time *t* in the acceptor well, *V*_D_ is the volume in the donor well, *t* is time in seconds, *A* is the area of the membrane in cm, and QD(*t*_0_) is the amount of compound in the donor compartment at the beginning of the experiment.

To analyze the integrity of the mAb after BBB crossing, 500 μL of Tz, Tz-Ang2, and Tz-MiniAp4 (5 μM) in ECM media was added to the donor compartment and 1500 μL of ECM media was introduced into the acceptor compartment. The plates were incubated for 16 h, but after 2 h, 500 μL of the acceptor compartment was removed for analysis and replaced with fresh media.

For MS analysis, proteins from the acceptor compartment were purified by immunoprecipitation with Protein A magnetic beads following the manufacturer’s instructions. In brief, 25 μL of beads was placed into a 1.5 mL microcentrifuge tube, diluted with PBST, and gently mixed. The tube was placed into a magnetic stand to facilitate the supernatant removal. 500 μL of PBS solution was added to the tube to wash the beads. After mixing, the solution was removed after collecting the beads with the magnetic stand. This operation was repeated three times. 1 mL of acceptor solution was added and left mixing with the beads o/n at 4 °C. Then, the supernatant was discarded and the beads were washed (3 × 500 μL PBST and 3 × 500 μL PBS). mAbs were eluted with 50 μL of 0.1 M glycine pH 2, and the solution was neutralized with 8 μL of 3 M Tris, pH 8.5. Samples were analyzed by LCT-MS.

#### Cell Culture

H2030-BrM and HCC1954-BrM1a were cultured in RPMI 1640 media supplemented with 10% FBS, 2 mM l-glutamine, 100 IU/mL penicillin/streptomycin, and 1 μg/mL amphotericin B.

#### Animal Studies

Biodistribution studies in mice were performed by ChemPartner animal facility according to protocols approved by the ChemPartner Institutional Animal Care and Use Committee (IACUC) following Assessment and Accreditation of Laboratory Animal Care (AAALAC) guidelines. CD-1 male mice (6–8 weeks) were injected with Tz, Tz-Ang2, or Tz-MiniAp4 (10 mg/kg) via tail vein injection. 8 h after injection, blood was collected for serum generation and brains were terminally collected. The amount of antibodies in brain tissue and serum was determined by ELISA (goat anti-human IgG F(c) Antibody, Sigma, Cat#609-101-017, Anti-Human IgG (Fab specific)-Peroxidase, Sigma, Cat#A0293).

All other animal experiments were performed in accordance with a protocol approved by the CNIO (IACUC.001-2020), Instituto de Salud Carlos III (CBA05_2020) and Comunidad de Madrid Institutional Animal Care and Use Committee (PROEX130.7/20). Athymic nu/nu (Harlan) 5–6 weeks of age were used. Brain colonization assays were performed by injecting 100 μL of PBS into the left ventricle containing 100,000 cancer cells. Brain colonization was analyzed in vivo and ex vivo by BLI. Anesthetized mice (isoflurane) were injected retro-orbitally with d-luciferin (150 mg/kg; Syd Laboratories) and imaged with an IVIS machine (PerkinElmer). Bioluminescence analysis was performed using Living Image software, version 4.5.

For the ASC accumulation in the metastatic brain experiment, mice were administered with Tz, Tz-Ang2, or Tz-MiniAp4 in PBS, as depicted in Figure 4a. For the reduction and prevention of breast cancer BM experiment, mice were administered with vehicle (10 mM NaOAc, 150 mM NaCl, and Tween 20 (86 μL/L) pH 5, Tz, or Tz-MiniAp4) following the protocol depicted in Figure 5a.

#### Organotypic Cultures

Organotypic cultures from adult mouse brain organs were dissected in HBSS supplemented with HEPES (pH 7.4, 2.5 mM), d-glucose (30 mM), CaCl_2_ (1 mM), MgCl_2_ (1 mM), and NaHCO_3_ (4 mM) and embedded in 4% low-melting agarose (Lonza) preheated at 42 °C. The embedded organs were cut into 250 μm slices using a vibratome (Leica). Brain slices were divided in the hemisphere into two pieces. Slices were placed with flat spatulas on top of 0.8 μm pore membranes (Sigma-Aldrich) floating on slice culture media (DMEM, supplemented HBSS, FBS 5%, l-glutamine (1 mM), and 100 IU/mL penicillin/streptomycin). Brain slices were imaged to confirm the presence of established metastases using BLI (day 0) and were cultured in the presence of the antibodies. Brain slices were imaged 3 days after (day 3).

#### Immunofluorescence

Tissue for immunofluorescence was obtained after overnight fixation with 4% PFA at 4 °C. Slicing of the brain was done by using a sliding microtome (Thermo Fisher Scientific). 80 μm was blocked in 10% NGS, 2% BSA, and 0.25% Triton X-100 in PBS for 2 h at room temperature (RT). Primary antibodies were incubated overnight at 4 °C in the blocking solution and the following day for 30 min at RT. After extensive washing in PBS–Triton 0.25%, the secondary antibody was added in the blocking solution and incubated for 2 h. After extensive washing in PBS–Triton 0.25%, nuclei were stained with bis-benzamide (1 mg/mL; Sigma-Aldrich) for 7 min at RT. Primary antibodies: GFP (1:1000; GFP-1020, Aves Laboratories). Secondary antibodies: Alexa-Fluor anti-chicken 488 (dilution 1:300; Invitrogen).

#### Image Acquisition and Analysis

Immunofluorescence images were acquired with a Leica SP5 upright confocal microscope at ×10 and ×20 objectives and analyzed with ImageJ software. Whole slides were acquired with a slide scanner (Axio Scan Z1, Zeiss).

#### Statistical Analysis

Data are represented as the mean ± the SEM unless otherwise indicated. Comparisons between two experimental groups were analyzed with unpaired, two-tailed Student’s *t* test.

## Results and Discussion

### Site-Specific Conjugation of the BBB-Shuttle Peptides to Trastuzumab

**Figure 1:**
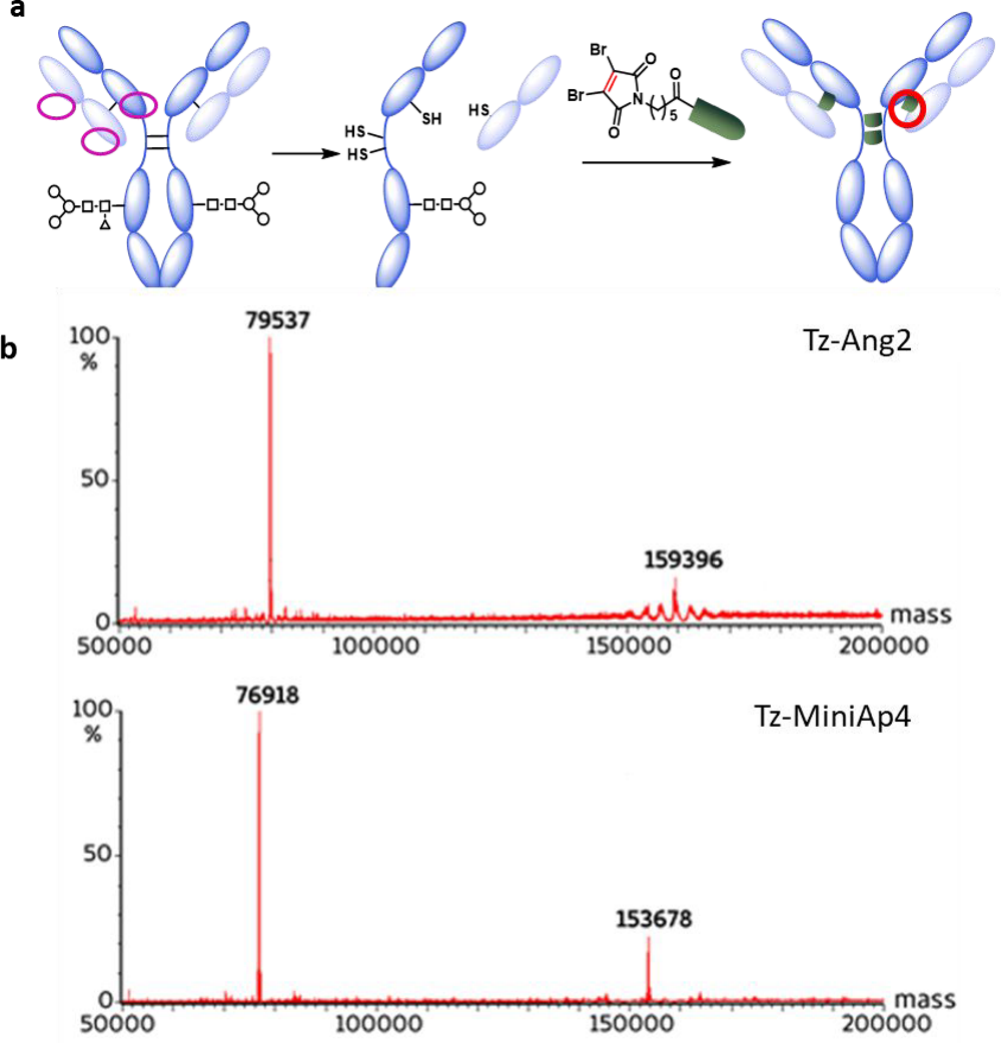
(a) ASC synthetic scheme. (b) Mass characterization of Tz-Ang2 (top) and Tz-MiniAp4 (bottom) by LCT-Premier. Deconvoluted spectra are shown. Mcal for Tz-Ang2 = 159,244; Mfound: 79,537, 159,396; Mcal for Tz-MiniAp4= 153,684; Mfound: 76,918, 153,678.

Given that the production of defined ADCs has been shown to maximize the therapeutic index, our first aim was to conjugate BBB shuttles to Tz in a site-specific manner. To this end, we set up an efficient chemical modification strategy that can be applied to full-length antibodies without genetic alterations. Modification of the interchain disulfide bridges with DBMs, also known as next-generation maleimides (NGMs), was selected due to the high serum stability of these compounds. Here, we broaden the scope of the reaction to peptides capable of mediating transport across biological barriers. The selected modification site does not alter the Fc structure of Tz in ADCs, minimizing the risk of affecting its effector functions.^48,49^ In addition, since the selected peptides are hydrophilic and do not contribute substantially to the charge or size of the antibodies, no difference between the aggregation propensity or clearance rate of Tz and Tz ASCs is expected.^50^ NGM was incorporated on the solid phase at the *N*-termini of the peptides (Scheme S1, Figures S1–S3, and Table S1). NGM-bearing brain shuttles were incorporated into the cysteines of the interchain disulfide bridges of trastuzumab upon reduction. Peptides were covalently linked by alkylation with the DBM peptides followed by incubation at pH 8.5, which led to hydrolysis of the maleimide group to yield the serum-stable maleamic acid (Figure 1 and Figures S4 and S5).

Incorporation of four peptides per antibody was confirmed by LC-MS and SDS-PAGE analyses, both for MiniA-p4 and for the control peptide Angiopep-2 (Figure 1 and Figure S4). The formation of only two isomers was observed, as previously reported,^46^ corresponding to the “full antibody” and “half antibody” resulting from the rebridging of the hinge cysteines in an intrachain mode (Figure 1 and Figure S5). Therefore, in contrast to previously reported ASCs,^41^ those prepared in this work are homogeneous, a highly desirable feature for a pharmacological product, enhancing the therapeutic index and facilitating product manufacturing and profiling. In addition, the incorporation of the selected peptide shuttles was achieved by taking advantage of the higher reactivity of the cysteines of the interchain disulfide bridges, thereby overcoming the need for genetic engineering to introduce a reactive tag and enabling the facile transfer of this strategy to other mAbs.

### *In Vitro* Functional Assessment of the Tz–BBB-Shuttle Conjugate

**Figure 2:**
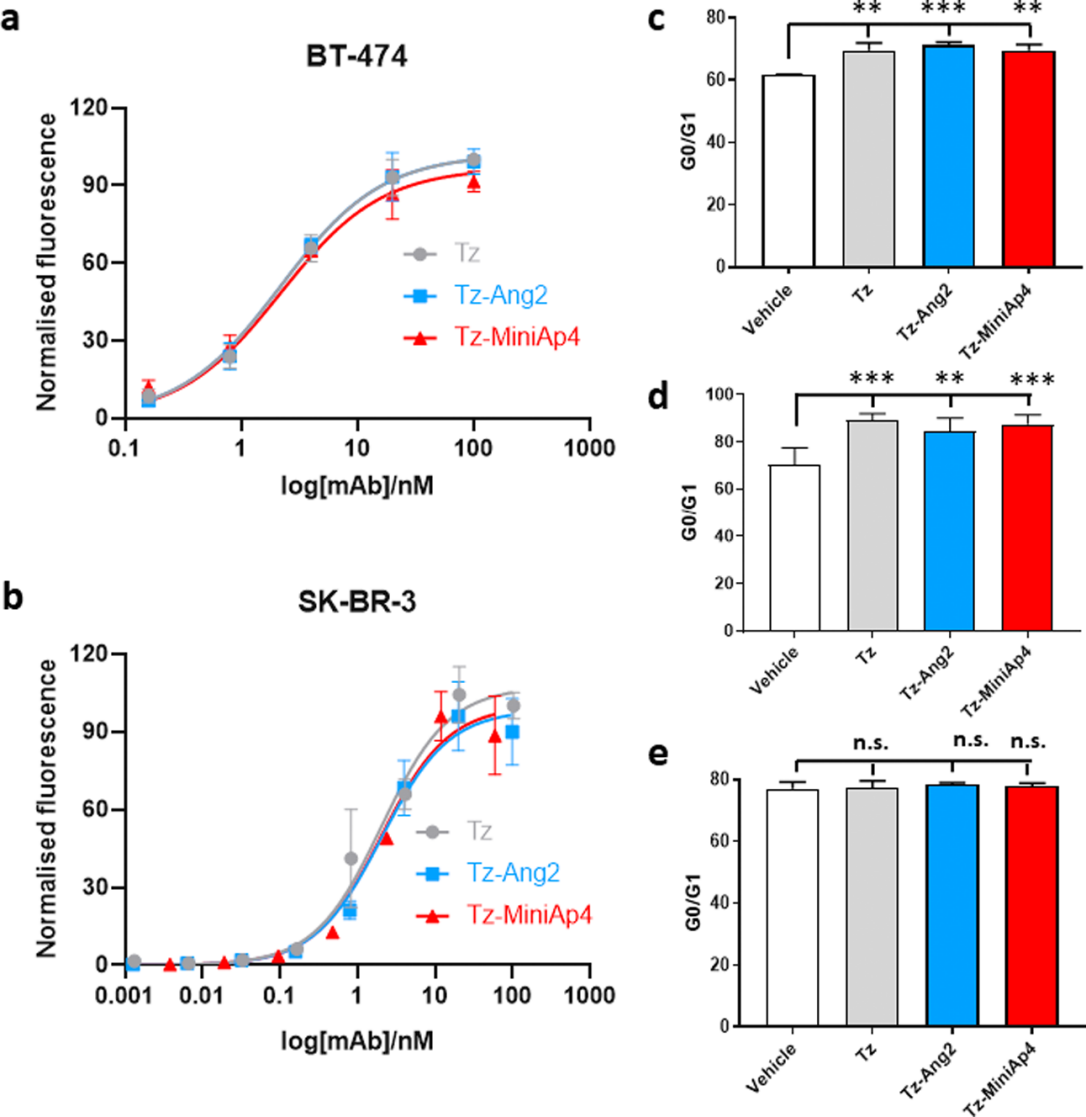
Binding of Tz, Tz-Ang2, or Tz-MiniAp4 to HER2-overexpressing cells: (a) BT-474; (b) SK-BR-3. Error bars represent the standard deviation (*n* = 3). Cell cycle arrest analysis of Tz-, Tz-Ang2-, or Tz-MiniAp4-treated cells: (c) SK-BR-3, (d) BT-474, and (e) MDA-MB-231. Error bars represent the standard deviation (*n* = 3). **p < 0.005; ***p < 0.0005.

We next verified that the antibody conjugates were fully functional upon modification. To this end, we first assessed their binding capacity and their ability to induce cell cycle arrest (Figure 2 and Figure S6). To determine affinity, we studied the binding to cells expressing high levels of the HER2/neu antigen. BT-474 breast ductal carcinoma and SK-BR-3 breast adenocarcinoma cells were incubated with different concentrations of modified and unmodified Tz at 4 °C, followed by flow cytometry analysis. This experiment showed that the binding capacity of Tz-Ang2 and Tz-MiniAp4 was identical to unmodified Tz in both cell lines (Figure 2a). In addition, confocal microscopy confirmed that the binding of modified and unmodified Tz was confirmed in BT-474 live cells. To this end, Tz was modified with a *N*-hydroxysuccinimide-modified far-red fluorophore (AF647) by the reaction of the solvent-exposed lysines. MS analysis confirmed the incorporation of and average of two fluorophores per antibody (Figure S4). MiniAp-4 or Angiopep-2 was then incorporated into the partially reduced antibody as previously detailed. After 1 h incubation with BT-474 cells at 4 °C, both modified and unmodified Tz was located at the cell membrane (Figure S8), as expected. These results confirm that the modification of Tz with the brain shuttles does not affect antibody binding. Subsequently, we evaluated the basis for the therapeutic properties of the Tz conjugates by measuring their ability to induce cell cycle arrest *in vitro*. The two BBB-shuttle-modified antibodies displayed cell cycle arrest properties similar to those of trastuzumab in HER2/neu+ cells (BT-474 and SK-BR-3) and had no effect on HER2/neu- MDA-MB-231 breast adenocarcinoma cells (Figure 2b and Figure S6).

### BBB Permeability in a Human-Cell-Based BBB Model

Having confirmed that the conjugates were functional, we sought to assess whether the brain shuttle peptides increased the transport of Tz in a human-cell-based BBB model.^47^ To this end, antibodies were radiolabeled with ^125^I and assayed at a low concentration (100 nM) to avoid receptor saturation.^24^ In this assay, MiniAp-4 improved Tz apparent permeability 3.4-fold, while Angiopep-2 enhanced transport only 2.5-fold (3a). In addition, evaluation of the stability of this peptide revealed a half-life of roughly 30 min (Figure S11).

**Figure 3:**
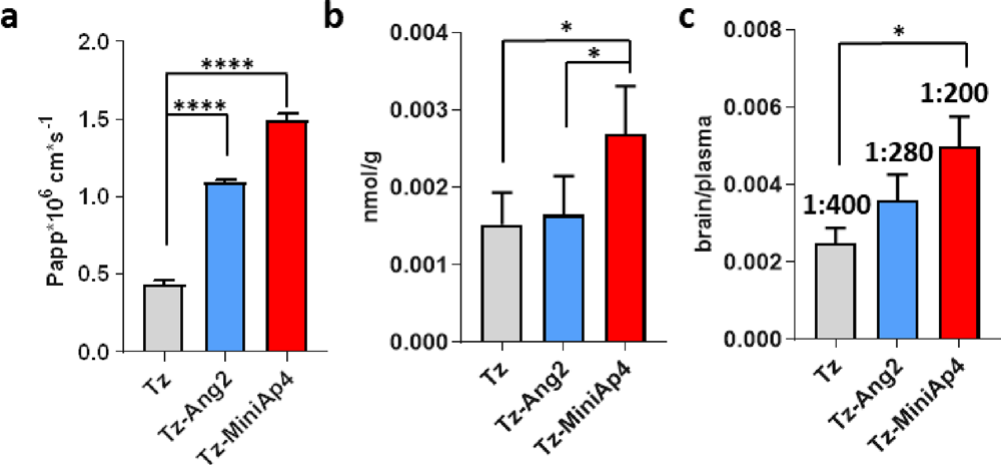
(a) Permeability of Tz, Tz-Ang2, and Tz-MiniAp4 (100 nM) in the human *in vitro* BBB cellular model. (b) Brain concentration of Tz, Tz-Ang2, and Tz-MiniAp4 after *i.v.* bolus injection. Results are expressed in terms of nmol/g of tissue. Error bars represent the standard deviation (*n* = 3). (c) Brain-to-plasma ratio. Results are expressed in terms of brain/serum ratio for mAbs. In all graphs, error bars represent the standard deviation (*n* = 3). *P* value was calculated using one-way ANOVA test. ****p < 0.001; *<0.05.

Since proteolytic activity at the BBB is very high, the integrity of the antibody conjugates was evaluated in the model, this time using a higher concentration of unlabeled conjugates (5 μM).^47^ The LC-MS confirmed that the main peaks corresponded to the intact constructs (Figure S9). However, upon closer examination, the mass spectra revealed for Tz-Ang2 was of lower quality and displayed additional peaks. These data suggest that Angiopep-2 might be partly degraded since it is a linear peptide composed by natural amino acids. Indeed, MALDI analysis of the acceptor revealed the proteolytic degradation of Ang2 after transport evaluation of the unmodified peptide in the BBB model (Figure S10).

While MiniAp-4 is also formed by l-amino acids, it has a cyclic structure with a lactam bridge that protects it from proteolysis, conferring it a half-life over 24 h.^31,32^ This property should have an even greater impact in *in vivo* experiments.

### ASC Accumulation in Healthy Mouse Brain

Encouraged by the BBB permeability results *in vitro*, we set out to confirm whether MiniAp-4 substantially enhanced the BBB transport of antibodies *in vivo*. To this end, mice were intravenously injected with a 10 mg/kg dose of each construct. After 8 h, saline was perfused to eliminate the antibodies in the blood or bound to the capillary lumen. The antibody concentration in serum and brain was measured by ELISA. The concentration of Tz in mouse brain was 225 ± 63 ng/g, and the brain/serum ratio was 1:400. These figures are consistent with previous reports.^41^ MiniAp-4 provided a twofold increase in both brain concentration and brain/serum ratio, while a small but nonsignificant increase was observed for Angiopep-2 (Figure 3b,c). The low efficiency of the latter in increasing the transport of Tz across the BBB transport might be attributable to its protease sensitivity and is in agreement with transport assays *in vitro.*(51)

### ASC Accumulation in Metastatic Mouse Brain

Subsequently, we sought to verify whether ASCs modified with BBB shuttles also increased the level of Tz accumulation in mice bearing brain metastases, which would be expected if the BBB was partly intact. To this end, athymic nude mice were inoculated intracardially with the model brain metastatic cell line H2030-BrM, leading to the development of metastasis. On day 25 postinoculation, once metastases were fully established as detected by noninvasive bioluminescence, AF647-labeled Tz, Tz-Ang2, and Tz-MiniAp4 antibodies were administered systemically (10 mg/kg, IC) and mice were sacrificed 4 h later. Brain accumulation of ASCs was assessed by near-infrared (NIR) imaging. The fluorescent intensity of mice treated with Tz-Ang2 and Tz-MiniAp4 was visibly higher than the one treated with Tz alone, suggesting a greater brain penetration of the BBB-shuttle-modified mAbs (4 and Figure S12).

**Figure 4:**
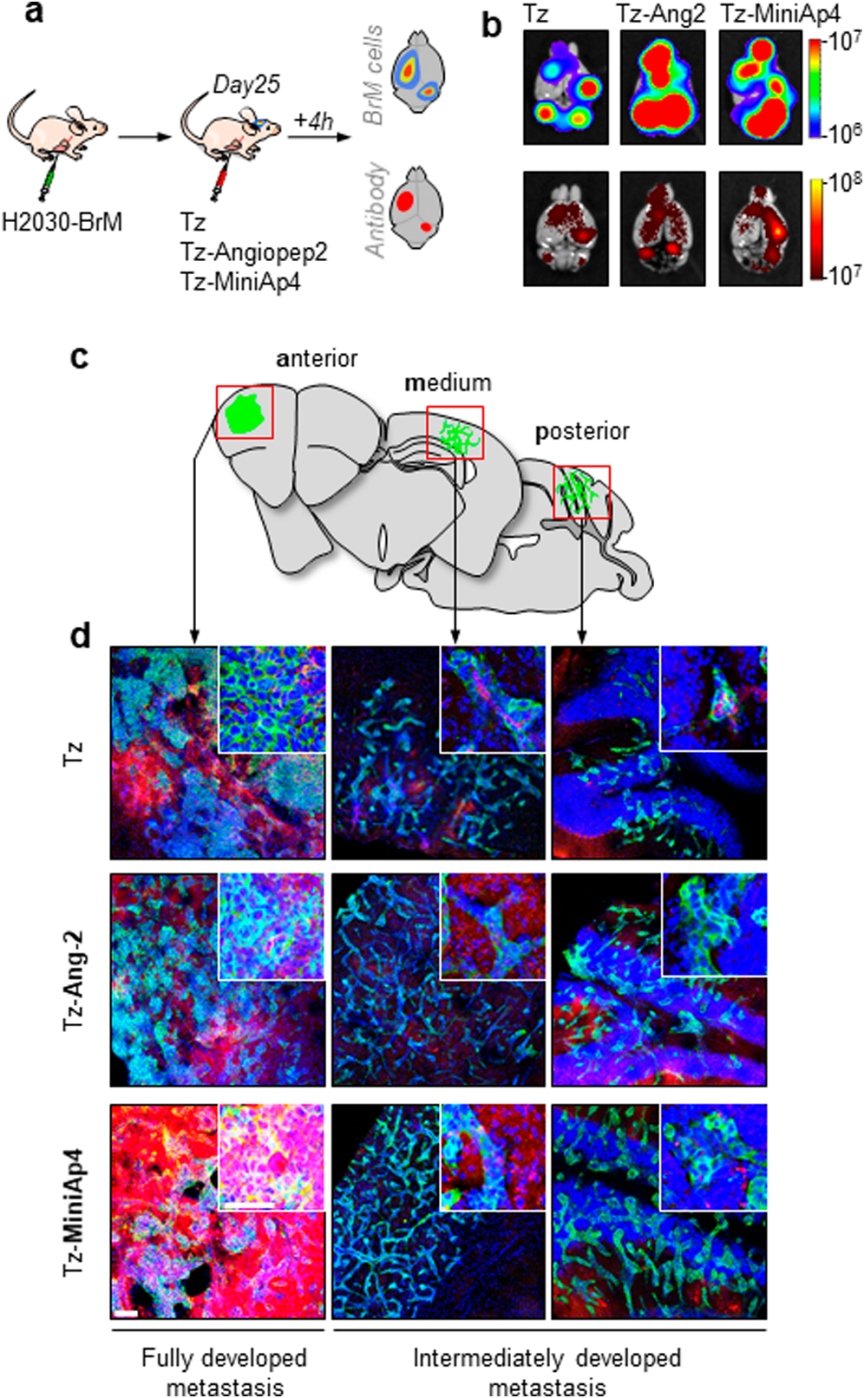
BBB-shuttle peptides allow for higher brain accumulation of Tz in metastatic mouse brains. (a) Schema of experimental design. Upon intracardial injection of the brain metastatic H2030-BrM cancer cell line expressing GFP and luciferase, the indicated compounds, labeled with NIR antibodies, were administered systemically and mice were killed 4 h later. (b) Representative *ex vivo* images of bioluminescence (cancer cells) and fluorescence (antibodies) in the brains of inoculated mice. (c) Three anterior-posterior levels of the cerebral cortex were analyzed by histology to measure fluorescence derived from antibody accumulation in areas affected by metastases. (d) Representative low and high magnifications of metastatic cells (green, GFP) and the various therapeutic antibodies (red, NIR antibodies). Blue, bis-benzamide. Scale bars: 100 μm, low magnification; 50 μm, high magnification.

In addition, confocal analysis of various regions of the brain (Bregma 1.70 mm-anterior-, −1.06 mm -medium-, −7.08 mm -posterior-) of treated mice revealed variation in the accumulation of fluorescent signals for each construct, with Tz-MiniAp4 showing the greatest capacity to enter the brain (Figure 4).

### Reduction and Prevention of Breast Cancer Brain Metastasis

The preliminary study in the murine model of BM encouraged us to study the potential of Tz-MiniAp4 to reduce and prevent metastasis, first *ex vivo* and then *in vivo*.

We first analyzed the effect of Tz and the ASC in METPlatform, an *ex vivo* drug-screening tool used to evaluate potential drugs for the treatment of metastases growing in the organs being colonized.^52,53^ In brief, athymic nude mice were injected intracardially with the HER2+ breast ductal carcinoma-brain metastatic cell line HCC1954-BrM1a^54^ until brain metastases were established. At that moment (25 days after injection), the brains including the metastases were obtained and processed into organotypic cultures.^52,53^ Next, Tz and Tz-MiniAp4 were added to the media at 10 and 100 μgmL^–1^ and after 3 days, their impact on the viability of metastases was measured by evaluating cancer-cell-derived bioluminescence, which was normalized by the specific bioluminescence levels present in each slice before addition of ASCs. As anticipated, treatment with both compounds reduced metastasis-derived bioluminescence in comparison with the control condition. Since the BBB does not limit the access of the mAb in METPlatform, no substantial differences in efficacy were observed between the naked Tz and the brain shuttle-modified Tz-MiniAp4 (Figure S12), thereby confirming that the evaluated ASCs are fully functional, as previously tested *in vitro* (Figure 2b).^55^ In addition, since modification of Tz did not affect its therapeutic performance, the ability of targeting circulating tumor cells in HER2-positive breast cancer reported for Tz would also be maintained for the newly prepared ASCs.^56,57^

**Figure 5:**
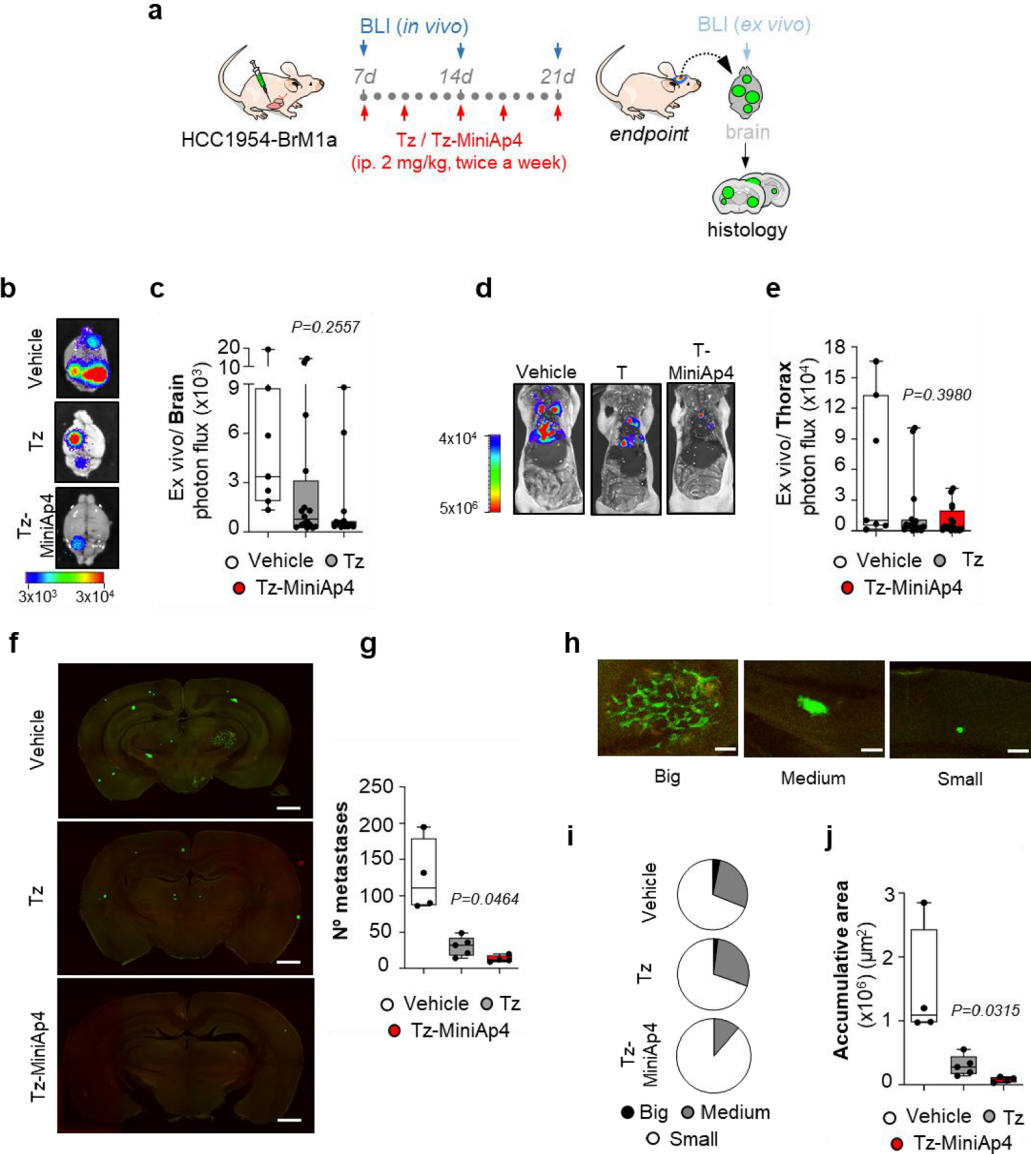
(a) Schema of the experimental design. (b) Representative images of brains obtained from mice at the end point of the experiment showing their bioluminescence signal. (c) Quantification of *ex vivo* bioluminescent images (BLI) of brains at the end point of the experiment (vehicle *n* = 7; Tz *n* = 16; Tz-MiniAp4 *n* = 16 mice per experimental condition). (d) Representative images of thorax obtained from mice at the end point of the experiment showing their bioluminescence signal. (e) Quantification of *ex vivo* BLI of thoracic regions at the end point of the experiment. (f) Representative sections of brains from vehicle-, Tz-, and Tz-Miniap4-treated mice. Scale bar, 1000 μm. (g) Quantification of the number of established metastases found in vehicle-, Tz-, and Tz-Miniap4-treated brains (vehicle *n* = 4; Tz *n* = 5; Tz-MiniAp4 *n* = 4 mice per experimental condition). (h) Representative images showing established metastases with different sizes (big, >50,000 μm^2^; medium, 10,000–50,000 μm^2^; and small, <10,000 μm^2^) at the end point of the experiment. Scale bars, 200 μm. (i) Quantification of the number of established metastases according to the size (big, medium, and small) in each experimental condition (vehicle, Tz, Tz-MiniAp4). In vehicle slices (*n* = 4), 3.4% were big, 27.6% were medium, and 69% were small. In Tz slices (*n* = 5), 2% were big, 28% were medium, and 70% were small. In Tz-MiniAp4-slices (*n* = 4), 0% were big, 11.5% were medium, and 88.5% were small. (j) Quantification of the accumulative area of established metastases found in vehicle-, Tz-, and Tz-Miniap4-treated brains (vehicle *n* = 4; Tz *n* = 5; Tz-MiniAp4 *n* = 4 mice per experimental condition). *P* value was calculated using two-tailed *t* test (vehicle vs Tz *p* = 0.0213; vehicle vs Tz-MiniAp4 *p* = 0.02; Tz vs Tz-MiniAp4 *p* = 0.0315).

Once we had confirmed that the ASC has the same capacity to reduce metastasis *ex vivo,* we addressed the final challenge, namely, preventing the development of brain metastases *in vivo* (5). To this end, mice inoculated with the HCC1954-BrM1a cell line were treated with 2 mg kg^–1^ Tz and Tz-MiniAp4 twice a week once metastatic cells have completed extravasated across the BBB (Figure 5a).^58^ Treatment was maintained for 2 weeks. After the fifth dose, the mice were sacrificed.^59^

This protocol was designed to mimic the clinical situation, thus aiming to treat brain metastases while still being not detectable. *Ex vivo* analysis of brains with bioluminescence showed a tendency of decreases metastases in both treatments (Figure 5b,c). The same tendency was observed after *ex vivo* analysis of the thoracic regions, as expected (Figure 5d,e). A detailed histological analysis offered a more accurate evaluation of metastatic colonization. Although both antibodies significantly decreased the total number of metastases (Figure 5f,g), only Tz-MiniAp4 reduced the formation of large metastases, including those 10,000 μm^2^ and larger, and fully prevented the presence of those above 50,000 μm^2^ (Figure 5h–j), which are the ones with immediate clinical interest. Overall, these data show that the conjugation of BBB-shuttle peptide MiniAp-4 to Tz provided a more efficacious preventive treatment for breast cancer metastasis to the brain.

## Conclusions

In summary, here, we developed a homogeneous antibody–brain shuttle conjugate capable of crossing the BBB and reducing the number and size of cancer metastases to the brain. We set up a simple and efficient conjugation method to covalently link DBM-bearing peptides with the reduced interchain disulfide bridges of a mAb. We applied this method to anchor MiniAp-4, a protease-resistant cyclic BBB-shuttle previously developed by our group, and Angiopep-2, a reference lineal BBB-shuttle peptide, to the anti-HER2 antibody Tz. Both conjugates are homogeneous and preserve a high affinity for the antigen. Although both MiniAp-4 and Angiopep-2 enhanced the transport of Tz in a human-cell-based model, the former increased permeability 3.5-fold while the second achieved only a 2.4-fold increase. Moreover, under the conditions assayed, only MiniAp-4 showed capacity to enhance the transport of Tz *in vivo*. Evaluation of the MiniAp4 ASC in a murine model of BM demonstrated that the higher brain accumulation of this conjugate resulted in an improved therapeutic effect with respect to the unmodified Tz, fully preventing the development of clinically relevant metastases. Since HER2+ breast cancer patients are in the highest risk group for developing BM,^17−19^ it is crucial to explore preventative strategies using new therapeutic agents. In this context, we propose that MiniAp-4 could significantly increase the therapeutic benefits of trastuzumab-based therapies. The preventive effect reported here would enable turning brain metastases from a terminal condition to a chronic disease. Large and medium metastases correlated with the poorest prognosis. The decrease in the number of these metastases observed under the treatment with Tz-MiniAp4 would enable controlling disease progression, thereby prolonging patients’ lifespans and improving their quality of life. Furthermore, this ASC technology has the potential to be extended to any antibody intended to treat CNS-related diseases, ranging from metastatic and primary brain cancers to neurodegenerative diseases.

## Supporting Information Available

^1^H and ^13^C NMR spectra of 3,4-dibromo-2,5-dioxo-2,5-dihydro-1*H*-pyrrol-1-yl)acetic acid; UPLC and MS data of DBM-Ang2 and DBM-miniAp4; Mw and isoelectric point of peptides and mAb conjugates; cell cycle arrest data of SKBR-3, BT-474, or MDA-MB-231 cells treated with Tz, Tz-Ang2, and Tz-MiniAp4; MS characterization of AF647-modified Tz, Tz-Ang2, and Tz-MiniAp4; confocal images of BT-474 cells incubated with Tz, Tz-Ang2, and Tz-MiniAp4; mass characterization of Tz, Tz-Ang2, and Tz-MiniAp4 after immunoprecipitation of the acceptor well from HBBBCMTA; stability of Ang2 and MiniAp-4 in mouse serum; quantification of NIR fluorescence each mouse injected with Tz, Tz-Ang2, and Tz-MiniAp4; and data related to organotypic cultures treated with Tz and Tz-MiniAp4 (PDF)

## Abbreviations

ADC: antibody–drug conjugate
ASC: antibody–brain shuttle conjugate
Ang2: Angiopep-2
BM: brain metastasis
BBB: blood–brain barrier
BTB: blood–tumor barrier
DBM: dibromomaleimide
LC-MS: liquid chromatography coupled to mass spectrometry
mAbs: monoclonal antibodies
NIR: near infrared
NGM: next-generation maleimides
SDS-PAGE: sodium dodecyl sulfate polyacrylamide electrophoresis
Tz: trastuzumab

## References

[ref1] Lockman P. R.; Mittapalli R. K.; Taskar K. S.; Rudraraju V.; Gril B.; Bohn K. A.; Adkins C. E.; Roberts A.; Thorsheim H. R.; Gaasch J. A.; Huang S.; Palmieri D.; Steeg P. S.; Smith Q. R. Heterogeneous Blood–Tumor Barrier Permeability Determines Drug Efficacy in Experimental Brain Metastases of Breast Cancer. Clin. Cancer Res. 2010, 16 (23), 5664 10.1158/1078-0432.CCR-10-1564. 20829328 PMC2999649

[ref2] Arvanitis C. D.; Ferraro G. B.; Jain R. K. The Blood–Brain Barrier and Blood–Tumour Barrier in Brain Tumours and Metastases. Nat. Rev. Cancer 2020, 20 (1), 26–41. 10.1038/s41568-019-0205-x. 31601988 PMC8246629

[ref3] Poduslo J. F.; Curran G. L.; Berg C. T. Macromolecular Permeability across the Blood-Nerve and Blood-Brain Barriers. Proc. Natl. Acad. Sci. U.S.A. 1994, 91 (12), 5705 10.1073/pnas.91.12.5705. 8202551 PMC44065

[ref4] St-Amour I.; Paré I.; Alata W.; Coulombe K.; Ringuette-Goulet C.; Drouin-Ouellet J.; Vandal M.; Soulet D.; Bazin R.; Calon F. Brain Bioavailability of Human Intravenous Immunoglobulin and Its Transport through the Murine Blood–Brain Barrier. J. Cereb. Blood Flow Metab. 2013, 33 (12), 1983–1992. 10.1038/jcbfm.2013.160. 24045402 PMC3851908

[ref5] Daneman R.; Prat A. The Blood-Brain Barrier. Cold Spring Harb. Perspect. Biol. 2015, 7 (1), a020412–a020412. 10.1101/cshperspect.a020412. 25561720 PMC4292164

[ref6] Abbott N. J.; Patabendige A. A. K.; Dolman D. E. M.; Yusof S. R.; Begley D. J. Structure and Function of the Blood–Brain Barrier. Neurobiol. Dis. 2010, 37 (1), 13–25. 10.1016/j.nbd.2009.07.030. 19664713

[ref7] Sweeney M. D.; Zhao Z.; Montagne A.; Nelson A. R.; Zlokovic B. V. Blood-Brain Barrier: From Physiology to Disease and Back. Physiol. Rev. 2019, 99 (1), 21–78. 10.1152/physrev.00050.2017. 30280653 PMC6335099

[ref8] Zhao Z.; Nelson A. R.; Betsholtz C.; Zlokovic B. V. Establishment and Dysfunction of the Blood-Brain Barrier. Cell 2015, 163 (5), 1064–1078. 10.1016/j.cell.2015.10.067. 26590417 PMC4655822

[ref9] Valiente M.; Ahluwalia M. S.; Boire A.; Brastianos P. K.; Goldberg S. B.; Lee E. Q.; Le Rhun E.; Preusser M.; Winkler F.; Soffietti R. The Evolving Landscape of Brain Metastasis. Trends in Cancer 2018, 4 (3), 176–196. 10.1016/j.trecan.2018.01.003. 29506669 PMC6602095

[ref10] Achrol A. S.; Rennert R. C.; Anders C.; Soffietti R.; Ahluwalia M. S.; Nayak L.; Peters S.; Arvold N. D.; Harsh G. R.; Steeg P. S.; Chang S. D. Brain Metastases. Nat. Rev. Dis. Prim. 2019, 5 (1), 5 10.1038/s41572-018-0055-y. 30655533

[ref11] Barnholtz-Sloan J. S.; Sloan A. E.; Davis F. G.; Vigneau F. D.; Lai P.; Sawaya R. E. Incidence Proportions of Brain Metastases in Patients Diagnosed (1973 to 2001) in the Metropolitan Detroit Cancer Surveillance System. J. Clin. Oncol. 2004, 22 (14), 2865–2872. 10.1200/JCO.2004.12.149. 15254054

[ref12] Rostami R.; Mittal S.; Rostami P.; Tavassoli F.; Jabbari B. Brain Metastasis in Breast Cancer: A Comprehensive Literature Review. J. Neurooncol. 2016, 127 (3), 407–414. 10.1007/s11060-016-2075-3. 26909695

[ref13] Kennecke H.; Yerushalmi R.; Woods R.; Cheang M. C. U.; Voduc D.; Speers C. H.; Nielsen T. O.; Gelmon K. Metastatic Behavior of Breast Cancer Subtypes. J. Clin. Oncol. 2010, 28 (20), 3271–3277. 10.1200/JCO.2009.25.9820. 20498394

[ref14] Martin A. M.; Cagney D. N.; Catalano P. J.; Warren L. E.; Bellon J. R.; Punglia R. S.; Claus E. B.; Lee E. Q.; Wen P. Y.; Haas-Kogan D. A.; Alexander B. M.; Lin N. U.; Aizer A. A. Brain Metastases in Newly Diagnosed Breast Cancer: A Population-Based Study. JAMA Oncol. 2017, 3 (8), 1069–1077. 10.1001/jamaoncol.2017.0001. 28301662 PMC5824221

[ref15] Pasquier D.; Darlix A.; Louvel G.; Fraisse J.; Jacot W.; Brain E.; Petit A.; Mouret-Reynier M. A.; Goncalves A.; Dalenc F.; Deluche E.; Fresnel J. S.; Augereau P.; Ferrero J. M.; Geffrelot J.; Fumet J.-D.; Lecouillard I.; Cottu P.; Petit T.; Uwer L.; Jouannaud C.; Leheurteur M.; Dieras V.; Robain M.; Mouttet-Audouard R.; Bachelot T.; Courtinard C. Treatment and Outcomes in Patients with Central Nervous System Metastases from Breast Cancer in the Real-Life ESME MBC Cohort. Eur. J. Cancer 2020, 125, 22–30. 10.1016/j.ejca.2019.11.001. 31835235

[ref16] Cavaco M.; Gaspar D.; Arb Castanho M.; Neves V. Antibodies for the Treatment of Brain Metastases, a Dream or a Reality?. Pharmaceutics 2020, 12 (1), 62 10.3390/pharmaceutics12010062. 31940974 PMC7023012

[ref17] Montemurro F.; Delaloge S.; Barrios C. H.; Wuerstlein R.; Anton A.; Brain E.; Hatschek T.; Kelly C. M.; Peña-Murillo C.; Yilmaz M.; Donica M.; Ellis P. Trastuzumab Emtansine (T-DM1) in Patients with HER2-Positive Metastatic Breast Cancer and Brain Metastases: Exploratory Final Analysis of Cohort 1 from KAMILLA, a Single-Arm Phase IIIb Clinical Trial. Ann. Oncol. 2020, 31 (10), 1350–1358. 10.1016/j.annonc.2020.06.020. 32634611

[ref18] Modi S.; Saura C.; Yamashita T.; Park Y. H.; Kim S.-B.; Tamura K.; Andre F.; Iwata H.; Ito Y.; Tsurutani J.; Sohn J.; Denduluri N.; Perrin C.; Aogi K.; Tokunaga E.; Im S.-A.; Lee K. S.; Hurvitz S. A.; Cortes J.; Lee C.; Chen S.; Zhang L.; Shahidi J.; Yver A.; Krop I. Trastuzumab Deruxtecan in Previously Treated HER2-Positive Breast Cancer. N. Engl. J. Med. 2020, 382 (7), 610–621. 10.1056/NEJMoa1914510. 31825192 PMC7458671

[ref19] Murthy R. K.; Loi S.; Okines A.; Paplomata E.; Hamilton E.; Hurvitz S. A.; Lin N. U.; Borges V.; Abramson V.; Anders C.; Bedard P. L.; Oliveira M.; Jakobsen E.; Bachelot T.; Shachar S. S.; Müller V.; Braga S.; Duhoux F. P.; Greil R.; Cameron D.; Carey L. A.; Curigliano G.; Gelmon K.; Hortobagyi G.; Krop I.; Loibl S.; Pegram M.; Slamon D.; Palanca-Wessels M. C.; Walker L.; Feng W.; Winer E. P. Tucatinib, Trastuzumab, and Capecitabine for HER2-Positive Metastatic Breast Cancer. N. Engl. J. Med. 2020, 382 (7), 597–609. 10.1056/NEJMoa1914609. 31825569

[ref20] Kinoshita M.; McDannold N.; Jolesz F. A.; Hynynen K. Targeted Delivery of Antibodies through the Blood–Brain Barrier by MRI-Guided Focused Ultrasound. Biochem. Biophys. Res. Commun. 2006, 340 (4), 1085–1090. 10.1016/j.bbrc.2005.12.112. 16403441

[ref21] Boado R. J.; Zhang Y.; Zhang Y.; Pardridge W. M. Humanization of Anti-Human Insulin Receptor Antibody for Drug Targeting across the Human Blood–Brain Barrier. Biotechnol. Bioeng. 2007, 96 (2), 381–391. 10.1002/bit.21120. 16937408

[ref22] Niewoehner J.; Bohrmann B.; Collin L.; Urich E.; Sade H.; Maier P.; Rueger P.; Stracke J. O.; Lau W.; Tissot A. C.; Loetscher H.; Ghosh A.; Freskgård P.-O. Increased Brain Penetration and Potency of a Therapeutic Antibody Using a Monovalent Molecular Shuttle. Neuron 2014, 81 (1), 49–60. 10.1016/j.neuron.2013.10.061. 24411731

[ref23] Kariolis M. S.; Wells R. C.; Getz J. A.; Kwan W.; Mahon C. S.; Tong R.; Kim D. J.; Srivastava A.; Bedard C.; Henne K. R.; Giese T.; Assimon V. A.; Chen X.; Zhang Y.; Solanoy H.; Jenkins K.; Sanchez P. E.; Kane L.; Miyamoto T.; Chew K. S.; Pizzo M. E.; Liang N.; Calvert M. E. K.; DeVos S. L.; Baskaran S.; Hall S.; Sweeney Z. K.; Thorne R. G.; Watts R. J.; Dennis M. S.; Silverman A. P.; Zuchero Y. J. Y. Brain Delivery of Therapeutic Proteins Using an Fc Fragment Blood-Brain Barrier Transport Vehicle in Mice and Monkeys. Sci. Transl. Med. 2020, 12 (545), eaay1359 10.1126/scitranslmed.aay1359. 32461332

[ref24] Demeule M.; Poirier J.; Jodoin J.; Bertrand Y.; Desrosiers R. R.; Dagenais C.; Nguyen T.; Lanthier J.; Gabathuler R.; Kennard M.; Jefferies W. A.; Karkan D.; Tsai S.; Fenart L.; Cecchelli R.; Béliveau R. High Transcytosis of Melanotransferrin (P97) across the Blood–Brain Barrier. J. Neurochem. 2002, 83 (4), 924–933. 10.1046/j.1471-4159.2002.01201.x. 12421365

[ref25] Oller-Salvia B.; Sánchez-Navarro M.; Giralt E.; Teixidó M. Blood-Brain Barrier Shuttle Peptides: An Emerging Paradigm for Brain Delivery. Chem. Soc. Rev. 2016, 45 (17), 4690–4707. 10.1039/C6CS00076B. 27188322

[ref26] Sánchez-Navarro M.; Giralt E.; Teixidó M. Blood–Brain Barrier Peptide Shuttles. Curr. Opin. Chem. Biol. 2017, 38, 134–140. 10.1016/j.cbpa.2017.04.019. 28558293

[ref27] Thom G.; Tian M.-M.; Hatcher J. P.; Rodrigo N.; Burrell M.; Gurrell I.; Vitalis T. Z.; Abraham T.; Jefferies W. A.; Webster C. I.; Gabathuler R. A Peptide Derived from Melanotransferrin Delivers a Protein-Based Interleukin 1 Receptor Antagonist across the BBB and Ameliorates Neuropathic Pain in a Preclinical Model. J. Cereb. Blood Flow Metab. 2019, 39 (10), 2074–2088. 10.1177/0271678X18772998. 29845881 PMC6775589

[ref200] Prades R.; Teixido M.; Oller-Salvia B. New trends in Brain Shuttle Peptides. Mol. Pharmaceutics 2025, 10.1021/acs.molpharmaceut.4c01327. PMC11881811 39899901

[ref28] Yu Y. J.; Zhang Y.; Kenrick M.; Hoyte K.; Luk W.; Lu Y.; Atwal J.; Elliott J. M.; Prabhu S.; Watts R. J.; Dennis M. S. Boosting Brain Uptake of a Therapeutic Antibody by Reducing Its Affinity for a Transcytosis Target. Sci. Transl. Med. 2011, 3 (84), 84ra44 LP-84ra44 10.1126/scitranslmed.3002230. 21613623

[ref29] Razpotnik R.; Novak N.; Čurin Šerbec V.; Rajcevic U. Targeting Malignant Brain Tumors with Antibodies. Front. Immunol. 2017, 1181 10.3389/fimmu.2017.01181. 28993773 PMC5622144

[ref30] Díaz-Perlas C.; Varese M.; Guardiola S.; García J.; Sánchez-Navarro M.; Giralt E.; Teixidó M. From Venoms to BBB-Shuttles. MiniCTX3: A Molecular Vector Derived from Scorpion Venom. Chem. Commun. 2018, 54 (90), 12738–12741. 10.1039/C8CC06725B. 30357254

[ref31] Oller-Salvia B.; Sánchez-Navarro M.; Ciudad S.; Guiu M.; Arranz-Gibert P.; Garcia C.; Gomis R. R.; Cecchelli R.; García J.; Giralt E.; Teixidó M. MiniAp-4: A Venom-Inspired Peptidomimetic for Brain Delivery. Angewandte Chemie International Edition 2016, 55 (2), 572–575. 10.1002/anie.201508445. 26492861 PMC4736446

[ref32] Fuster C.; Varese M.; García J.; Giralt E.; Sánchez-Navarro M.; Teixidó M. Expanding the MiniAp-4 BBB-Shuttle Family: Evaluation of Proline Cis-Trans Ratio as Tool to Fine-Tune Transport. J. Pept. Sci. 2019, 25 (5), e3172 10.1002/psc.3172. 31006945

[ref33] Díaz-Perlas C.; Sánchez-Navarro M.; Oller-Salvia B.; Moreno M.; Teixidó M.; Giralt E. Phage Display as a Tool to Discover Blood–Brain Barrier (BBB)-Shuttle Peptides: Panning against a Human BBB Cellular Model. Biopolymers 2017, 108 (1), e22928 10.1002/bip.22928. 27486695

[ref34] Guixer B.; Arroyo X.; Belda I.; Sabidó E.; Teixidó M.; Giralt E. Chemically Synthesized Peptide Libraries as a New Source of BBB Shuttles. Use of Mass Spectrometry for Peptide Identification. J. Pept. Sci. 2016, 22 (9), 577–591. 10.1002/psc.2900. 27440580

[ref35] Lucana M. C.; Lucchi R.; Gosselet F.; Díaz-Perlas C.; Oller-Salvia B. BrainBike Peptidomimetic Enables Efficient Transport of Proteins across Brain Endothelium. RSC Chem. Biol. 2024, 5 (1), 7–11. 10.1039/D3CB00194F. 38179197 PMC10763564

[ref36] Sawyers C. L. Herceptin: A First Assault on Oncogenes That Launched a Revolution. Cell 2019, 179 (1), 8–12. 10.1016/j.cell.2019.08.027. 31519311

[ref37] Costa R. L. B.; Czerniecki B. J. Clinical Development of Immunotherapies for HER2+ Breast Cancer: A Review of HER2-Directed Monoclonal Antibodies and Beyond. npj Breast Cancer 2020, 6 (1), 10 10.1038/s41523-020-0153-3. 32195333 PMC7067811

[ref38] Dijkers E. C.; Oude Munnink T. H.; Kosterink J. G.; Brouwers A. H.; Jager P. L.; de Jong J. R.; van Dongen G. A.; Schröder C. P.; Lub-de Hooge M. N.; de Vries E. G. Biodistribution of 89Zr-Trastuzumab and PET Imaging of HER2-Positive Lesions in Patients With Metastatic Breast Cancer. Clin. Pharmacol. Ther. 2010, 87 (5), 586–592. 10.1038/clpt.2010.12. 20357763

[ref39] Tamura K.; Kurihara H.; Yonemori K.; Tsuda H.; Suzuki J.; Kono Y.; Honda N.; Kodaira M.; Yamamoto H.; Yunokawa M.; Shimizu C.; Hasegawa K.; Kanayama Y.; Nozaki S.; Kinoshita T.; Wada Y.; Tazawa S.; Takahashi K.; Watanabe Y.; Fujiwara Y. 64Cu-DOTA-Trastuzumab PET Imaging in Patients with HER2-Positive Breast Cancer. J. Nucl. Med. 2013, 54 (11), 1869–1875. 10.2967/jnumed.112.118612. 24029656

[ref40] Demeule M.; Régina A.; Ché C.; Poirier J.; Nguyen T.; Gabathuler R.; Castaigne J. P.; Béliveau R. Identification and Design of Peptides as a New Drug Delivery System for the Brain. J. Pharmacol. Exp. Ther. 2008, 324 (3), 1064–1072. 10.1124/jpet.107.131318. 18156463

[ref41] Regina A.; Demeule M.; Tripathy S.; Lord-Dufour S.; Currie J. C.; Iddir M.; Annabi B.; Castaigne J. P.; Lachowicz J. E. ANG4043, a Novel Brain-Penetrant Peptide–MAb Conjugate, Is Efficacious against HER2-Positive Intracranial Tumors in Mice. Mol. Cancer Ther. 2015, 14 (1), 129–140. 10.1158/1535-7163.MCT-14-0399. 25492620

[ref42] Anami Y.; Xiong W.; Yamaguchi A.; Yamazaki C. M.; Zhang N.; An Z.; Tsuchikama K. Homogeneous Antibody–Angiopep 2 Conjugates for Effective Brain Targeting. RSC Adv. 2022, 12 (6), 3359–3364. 10.1039/D1RA08131D. 35425350 PMC8979263

[ref43] Behrens C. R.; Ha E. H.; Chinn L. L.; Bowers S.; Probst G.; Fitch-Bruhns M.; Monteon J.; Valdiosera A.; Bermudez A.; Liao-Chan S.; Wong T.; Melnick J.; Theunissen J.-W.; Flory M. R.; Houser D.; Venstrom K.; Levashova Z.; Sauer P.; Migone T.-S.; van der Horst E. H.; Halcomb R. L.; Jackson D. Y. Antibody–Drug Conjugates (ADCs) Derived from Interchain Cysteine Cross-Linking Demonstrate Improved Homogeneity and Other Pharmacological Properties over Conventional Heterogeneous ADCs. Mol. Pharmaceutics 2015, 12 (11), 3986–3998. 10.1021/acs.molpharmaceut.5b00432. PMC7755308 26393951

[ref44] Kaiser E.; Colescott R. L.; Bossinger C. D.; Cook P. I. Color Test for Detection of Free Terminal Amino Groups in the Solid-Phase Synthesis of Peptides. Anal. Biochem. 1970, 34 (2), 595–598. 10.1016/0003-2697(70)90146-6. 5443684

[ref45] Gasteiger E.; Hoogland C.; Gattiker A.; Duvaud S.; Wilkins M. R.; Appel R. D.; Bairoch A. Protein Identification and Analysis Tools on the ExPASy Server BT- The Proteomics Protocols Handbook; Walker J. M., Ed.; Humana Press: Totowa, NJ, 2005; pp 571–607. 10.1385/1-59259-890-0:571.

[ref46] Morais M.; Nunes J. P. M.; Karu K.; Forte N.; Benni I.; Smith M. E. B.; Caddick S.; Chudasama V.; Baker J. R. Optimisation of the Dibromomaleimide (DBM) Platform for Native Antibody Conjugation by Accelerated Post-Conjugation Hydrolysis. Org. Biomol. Chem. 2017, 15 (14), 2947–2952. 10.1039/C7OB00220C. 28290574

[ref47] Cecchelli R.; Aday S.; Sevin E.; Almeida C.; Culot M.; Dehouck L.; Coisne C.; Engelhardt B.; Dehouck M.-P.; Ferreira L. A Stable and Reproducible Human Blood-Brain Barrier Model Derived from Hematopoietic Stem Cells. PLoS One 2014, 9 (6), e99733 10.1371/journal.pone.0099733. 24936790 PMC4061029

[ref48] Junttila T. T.; Li G.; Parsons K.; Phillips G. L.; Sliwkowski M. X. Trastuzumab-DM1 (T-DM1) Retains All the Mechanisms of Action of Trastuzumab and Efficiently Inhibits Growth of Lapatinib Insensitive Breast Cancer. Breast Cancer Res. Treat. 2011, 128 (2), 347–356. 10.1007/s10549-010-1090-x. 20730488

[ref49] Ogitani Y.; Aida T.; Hagihara K.; Yamaguchi J.; Ishii C.; Harada N.; Soma M.; Okamoto H.; Oitate M.; Arakawa S.; Hirai T.; Atsumi R.; Nakada T.; Hayakawa I.; Abe Y.; Agatsuma T. DS-8201a, A Novel HER2-Targeting ADC with a Novel DNA Topoisomerase I Inhibitor, Demonstrates a Promising Antitumor Efficacy with Differentiation from T-DM1. Clin. Cancer Res. 2016, 22 (20), 5097–5108. 10.1158/1078-0432.CCR-15-2822. 27026201

[ref50] Lyon R. P.; Bovee T. D.; Doronina S. O.; Burke P. J.; Hunter J. H.; Neff-LaFord H. D.; Jonas M.; Anderson M. E.; Setter J. R.; Senter P. D. Reducing Hydrophobicity of Homogeneous Antibody-Drug Conjugates Improves Pharmacokinetics and Therapeutic Index. Nat. Biotechnol. 2015, 33 (7), 733–735. 10.1038/nbt.3212. 26076429

[ref51] Dumontet C.; Reichert J. M.; Senter P. D.; Lambert J. M.; Beck A. Antibody–Drug Conjugates Come of Age in Oncology. Nat. Rev. Drug Discovery 2023, 22 (8), 641–661. 10.1038/s41573-023-00709-2. 37308581

[ref52] Zhu L.; Retana D.; García-Gómez P.; Álvaro-Espinosa L.; Priego N.; Masmudi-Martín M.; Yebra N.; Miarka L.; Hernández-Encinas E.; Blanco-Aparicio C.; Martínez S.; Sobrino C.; Ajenjo N.; Artiga M.; Ortega-Paino E.; Torres-Ruiz R.; Rodríguez-Perales S.; Soffietti R.; Bertero L.; Cassoni P.; Weiss T.; Muñoz J.; Sepúlveda J. M.; González-León P.; Jiménez-Roldán L.; Moreno L. M.; Esteban O.; Pérez-Núñez Á.; Hernández-Laín A.; Toldos O.; Ruano Y.; Alcázar L.; Blasco G.; Fernández-Alén J.; Caleiras E.; Lafarga M.; Megías D.; Graña-Castro O.; Nör C.; Taylor M. D.; Young L. S.; Varešlija D.; Cosgrove N.; Couch F. J.; Cussó L.; Desco M.; Mouron S.; Quintela-Fandino M.; Weller M.; Pastor J.; Valiente M.; de la Lama-Zaragoza A.; Calero-Felix L.; Fiaño-Valverde C.; Delgado-López P. D.; Montalvo-Afonso A.; Pascual-Llorente M.; Díaz-Piqueras Á.; Nam-Cha S. H.; Barrena López C.; Plans Ahicart G.; Martínez-Saez E.; Ramón y Cajal S.; Nicolás P. A Clinically Compatible Drug-Screening Platform Based on Organotypic Cultures Identifies Vulnerabilities to Prevent and Treat Brain Metastasis. EMBO Mol. Med. 2022, 14 (3), e14552 10.15252/emmm.202114552. 35174975 PMC8899920

[ref53] Zhu L.; Miarka L.; Baena P.; Perea-García M.; Valiente M. Protocol to Generate Murine Organotypic Brain Cultures for Drug Screening and Evaluation of Anti-Metastatic Efficacy. STAR Protoc. 2023, 4 (2), 102194 10.1016/j.xpro.2023.102194. 37031412 PMC10120397

[ref54] Malladi S.; Macalinao D. G.; Jin X.; He L.; Basnet H.; Zou Y.; de Stanchina E.; Massagué J. Metastatic Latency and Immune Evasion through Autocrine Inhibition of WNT. Cell 2016, 165 (1), 45–60. 10.1016/j.cell.2016.02.025. 27015306 PMC4808520

[ref55] Gutierrez C.; Schiff R. HER2: Biology, Detection, and Clinical Implications. Arch. Pathol. Lab. Med. 2011, 135 (1), 55–62. 10.5858/2010-0454-RAR.1. 21204711 PMC3242418

[ref56] Zhang J.-L.; Yao Q.; Chen Y.; Wang J.-H.; Wang H.; Fan Q.; Ling R.; Yi J.; Wang L. Effects of Herceptin on Circulating Tumor Cells in HER2 Positive Early Breast Cancer. Genet. Mol. Res. 2015, 14 (1), 2099–2103. 10.4238/2015.March.20.20. 25867356

[ref57] Georgoulias V.; Bozionelou V.; Agelaki S.; Perraki M.; Apostolaki S.; Kallergi G.; Kalbakis K.; Xyrafas A.; Mavroudis D. Trastuzumab Decreases the Incidence of Clinical Relapses in Patients with Early Breast Cancer Presenting Chemotherapy-Resistant CK-19mRNA-Positive Circulating Tumor Cells: Results of a Randomized Phase II Study. Ann. Oncol. Off. J. Eur. Soc. Med. Oncol. 2012, 23 (7), 1744–1750. 10.1093/annonc/mds020. 22377561

[ref58] Valiente M.; Obenauf A. C.; Jin X.; Chen Q.; Zhang X. H.-F.; Lee D. J.; Chaft J. E.; Kris M. G.; Huse J. T.; Brogi E.; Massagué J. Serpins Promote Cancer Cell Survival and Vascular Co-Option in Brain Metastasis. Cell 2014, 156 (5), 1002–1016. 10.1016/j.cell.2014.01.040. 24581498 PMC3988473

[ref59] Eguren-Santamaria I.; Sanmamed M. F.; Goldberg S. B.; Kluger H. M.; Idoate M. A.; Lu B. Y.; Corral J.; Schalper K. A.; Herbst R. S.; Gil-Bazo I. PD-1/PD-L1 Blockers in NSCLC Brain Metastases: Challenging Paradigms and Clinical Practice. Clin. Cancer Res. 2020, 26 (16), 4186–4197. 10.1158/1078-0432.CCR-20-0798. 32354698

