# Supplementary material for: A Site-Specific MiniAp4–Trastuzumab Conjugate Prevents Brain Metastasis
Source: Mol Pharm. 2025 Feb 10;22(3):1384–95. doi: 10.1021/acs.molpharmaceut.4c01091 (PMC11881140; doi:10.1021/acs.molpharmaceut.4c01091)
Supplement: Supplementary file 1 — mp4c01091_si_001.pdf [file mp4c01091_si_001.pdf]

Supplementary information for

A site-specific MiniAp4-trastuzumab conjugate prevents brain metastasis

Mariam Masmudi-Martín, Benjamí Oller-Salvia, María Perea, Meritxell Teixidó, Manuel Valiente, Ernest Giralt, Macarena Sánchez-Navarro*

Mariam Masmudi-Martín – Brain Metastasis Group, CNIO, 28029 Madrid, Spain.

Benjamí Oller-Salvia - Institute for Research in Biomedicine (IRB Barcelona), Barcelona Institute of Science and Technology (BIST), 08028 Barcelona, Spain; Grup d'Enginyeria de Materials, Institut Químic de Sarrià (IQS), Universitat Ramon Llull, 08017 Barcelona, Spain.

María Perea – Brain Metastasis Group, CNIO, 28029 Madrid, Spain.

Meritxell Teixidó - Institute for Research in Biomedicine (IRB Barcelona), Barcelona Institute of Science and Technology (BIST), 08028 Barcelona, Spain. Present address: Gate2Brain, S. L. Baldiri Reixac 4-8, 08028Barcelona, Spain.

Manuel Valiente – Brain Metastasis Group, CNIO, 28029 Madrid, Spain.

Ernest Giralt - Institute for Research in Biomedicine (IRB Barcelona), Barcelona Institute of Science and Technology (BIST), 08028 Barcelona, Spain; Department of Inorganic and Organic Chemistry, University of Barcelona, 08028 Barcelona, Spain.

Macarena Sánchez Navarro* - Institute for Research in Biomedicine (IRB Barcelona), Barcelona Institute of Science and Technology (BIST), 08028 Barcelona, Spain; Department of Biochemistry and Molecular Pharmacology, Instituto de Parasitología y Biomedicina "López-Neyra" (CSIC), 18016 Granada, Spain; orcid.org/0000-0002-0159-2381; E-mail: macarena.sanchez@ipb.csic.es; Tel: 0034 958181660

Author Contributions: B. O.-S., M.T., E.G., M.V. and M.S.-N. designed the research; M. M.-M, M.P., B.O.-S. and M.S.-N. performed the research. All authors analysed the data; M.S.-N wrote the article with inputs from all the authors.

Competing Interest Statement: The authors declare no competing interest.

Keywords: BBB-shuttle; trastuzumab; brain metastases.

Scheme, figures and tables.

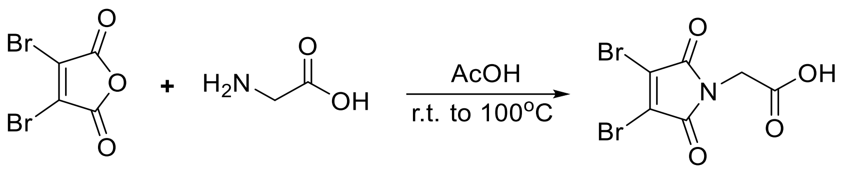

Scheme S1. Synthesis of 3,4-dibromo-2,5-dioxo-2,5-dihydro-1H-pyrrol-1-yl)acetic acid.

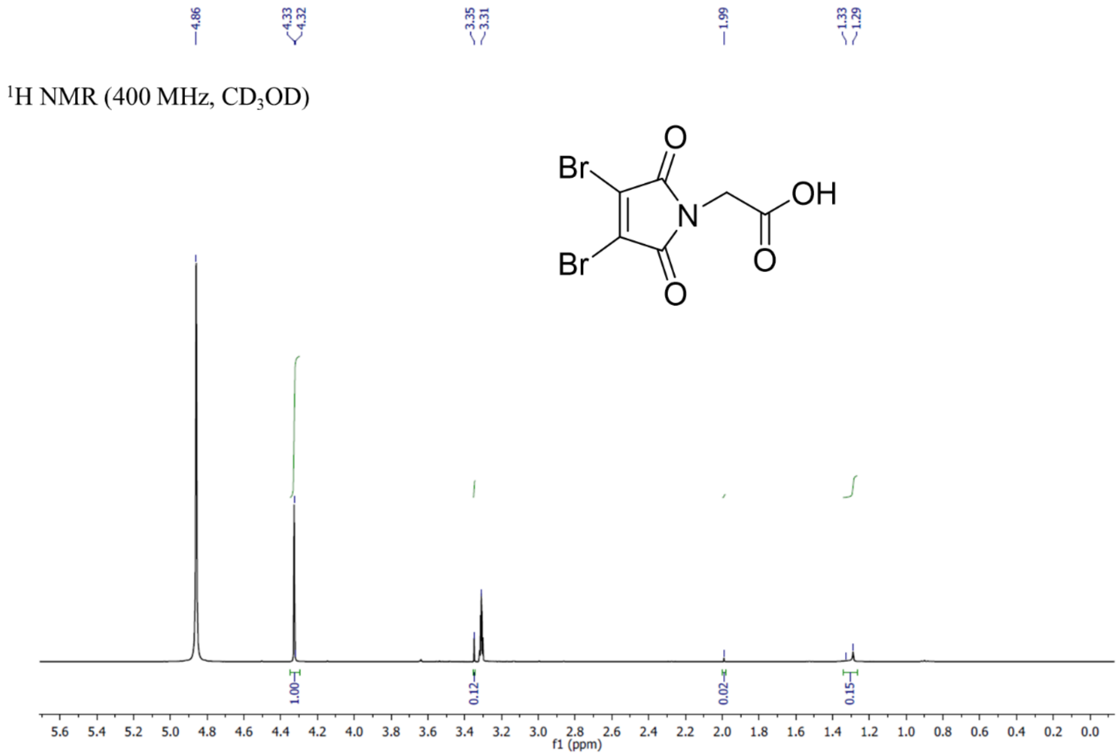

Figure S1. ^{1}H NMR of 3,4-dibromo-2,5-dioxo-2,5-dihydro-1H-pyrrol-1-yl)acetic acid.

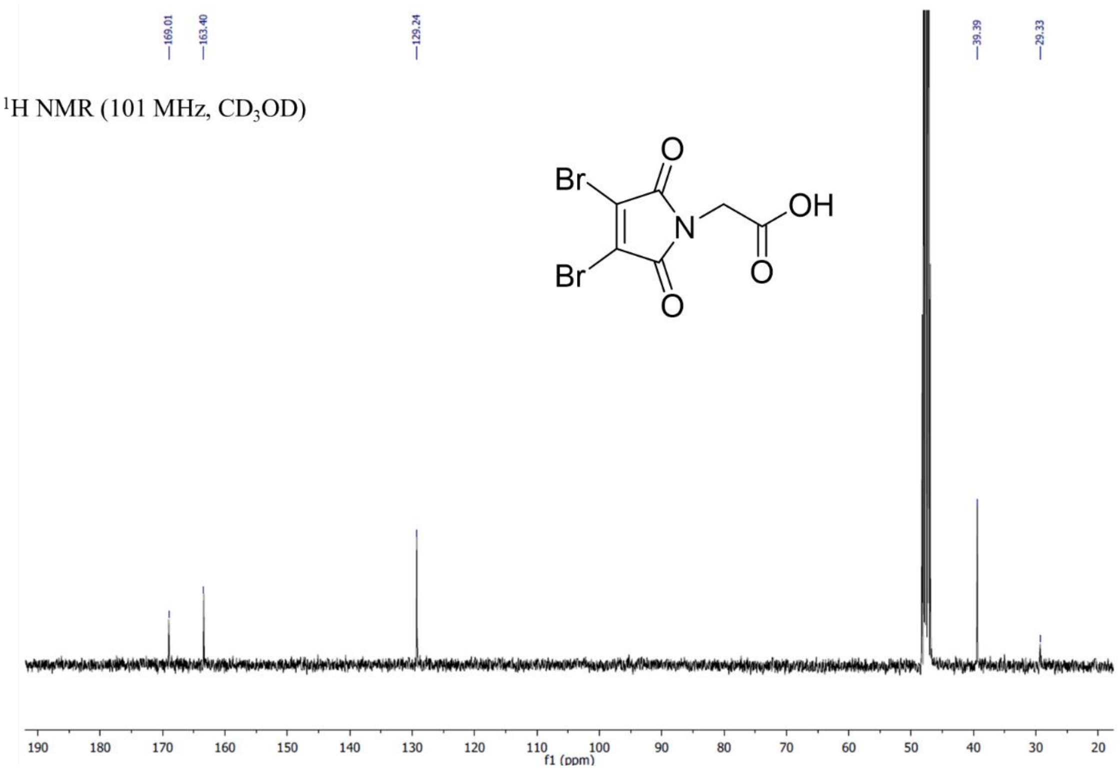

Figure S2. ^{13}C NMR of 3,4-dibromo-2,5-dioxo-2,5-dihydro-1H-pyrrol-1-yl)acetic acid.

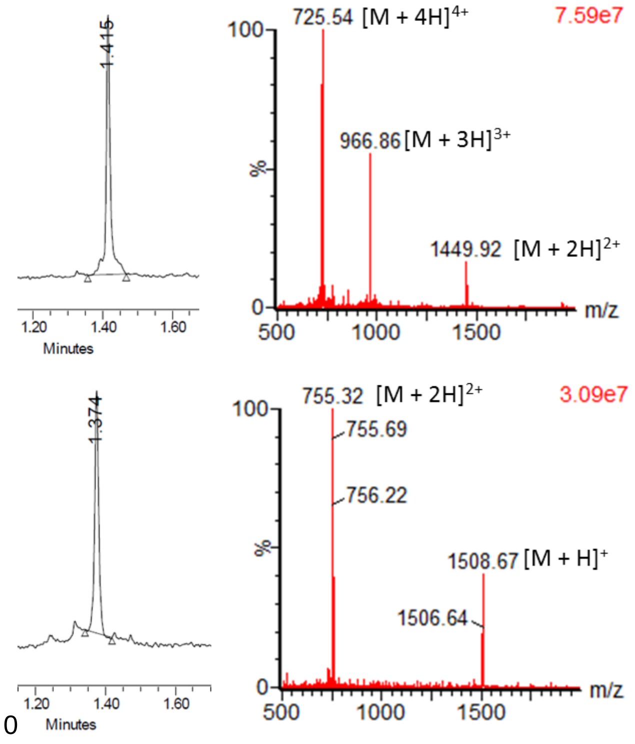

Figure S3. UPLC traces and MS spectra of the two peptides described in this paper. UPLC chromatograms are recorded at 220 nm in a 2-min linear gradient from 0 to 100% of MeCN (0.036% TFA) in H_2O (0.045% TFA).

Table S1: Sequence, molecular formula, high-resolution molecular weight (MW), UPLC characterisation and purity after synthesis and purification of DBM-Ang2 and DBM-MiniAp4. (&) refers to cyclic peptides. Nomenclature is adapted from Spengler *et al*.[1]

Peptide	Molecular formula	Calc MW, Da	Found MW, Da	t_R UPLC, min	Purity, %	Sequence
**DBM-Ang2**	$C_{124}H_{176}Br_2N_{32}O_{39}$	2895,1139	2895.1296	1.415	>95	DBM-TTDS-TFFYGGSRGKRNNFKTEEY-OH
**DBM-MiniAp4**	$C_{59}H_{94}Br_2N_{14}O_{22}$	1508.5034	1508.4992	1.374	>95	DBM-TTDS-[Dap](&)KAPETALD(&)-NH_2

Table S2: Mw and isoelectric point (pI) DBM-Ang2, DBM-miniAp4, Tz and the ASC Tz-MiniAp4 and Tz-Ang2.

Compound	Mw/Da	pI[a]
**DBM-TTDS-TFFYGGSRGKRNNFKTEEY-OH**	2895.11	9.52
**DBM-TTDS-[Dap](&)KAPETALD(&)-NH_2**	1508.50	4.37
**Tz**	148212	8.36
**Tz-MiniAp4**	153684	8.35
**Tz-Ang2**	159244	8.66

[a]pI was calculated using ProtParam Tool from expasy: Gasteiger E., Hoogland C., Gattiker A., Duvaud S., Wilkins M.R., Appel R.D., Bairoch A.; Protein Identification and Analysis Tools on the Expasy Server; (In) John M. Walker (ed): The Proteomics Protocols Handbook, Humana Press (2005) pp. 571-607.

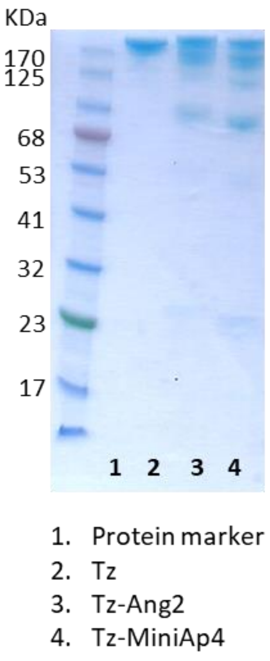

Figure S4. Coomassie-stained SDS-PAGE for the Tz conjugates.

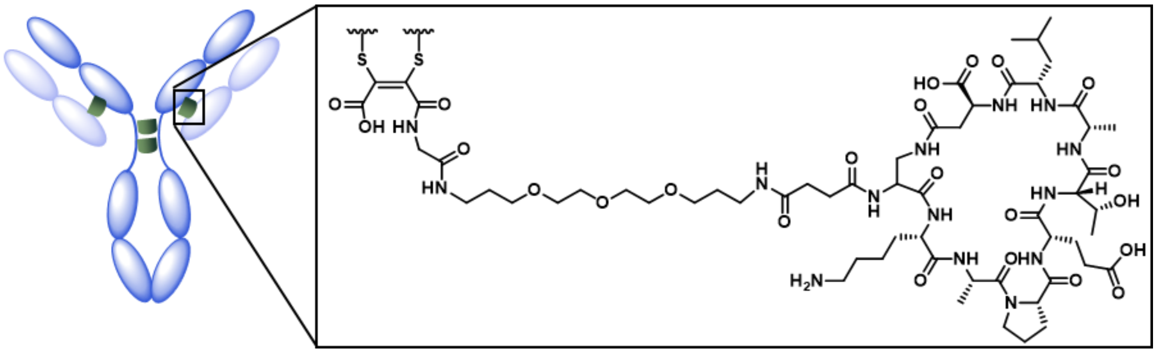

Figure S5. Schematic representation of Tz-MiniAp4 conjugate

SK-BR-3	% of cells in G_0/G_1
Vehicle	61.7 ± 0.2
Tz	69.5 ± 2.5
Tz-Ang2	71. 2 ± 1.0
Tz-MiniAp4	69.4 ± 1.9

BT-474	% of cells in G_0/G_1
Vehicle	70.5 ± 6.9
Tz	89.1 ± 2.8
Tz-Ang2	84.5 ± 5.8
Tz-MiniAp4	87.6 ± 3.8

MDA-MB-231	% of cells in G_0/G_1
Vehicle	77.0 ± 2.2
Tz	77.4 ± 2.3
Tz-Ang2	78.4 ± 0.5
Tz-MiniAp4	78.8 ± 0.8

Figure S6. Cell cycle arrest analysis of Tz, Tz-Ang2 or Tz-MiniAp4 treated cells. SK-BR-3 (a), BT-474 (b) or MDA-MB-231 (c) cells were serum-starved and stimulated with Tz, Tz-Ang2 or Tz-MiniAp4 (100 nM) for 5 days. Cells were stained with propidium iodide and cell cycle was analysed by flow cytometry. Data are shown as mean ± standard deviation, n=3.

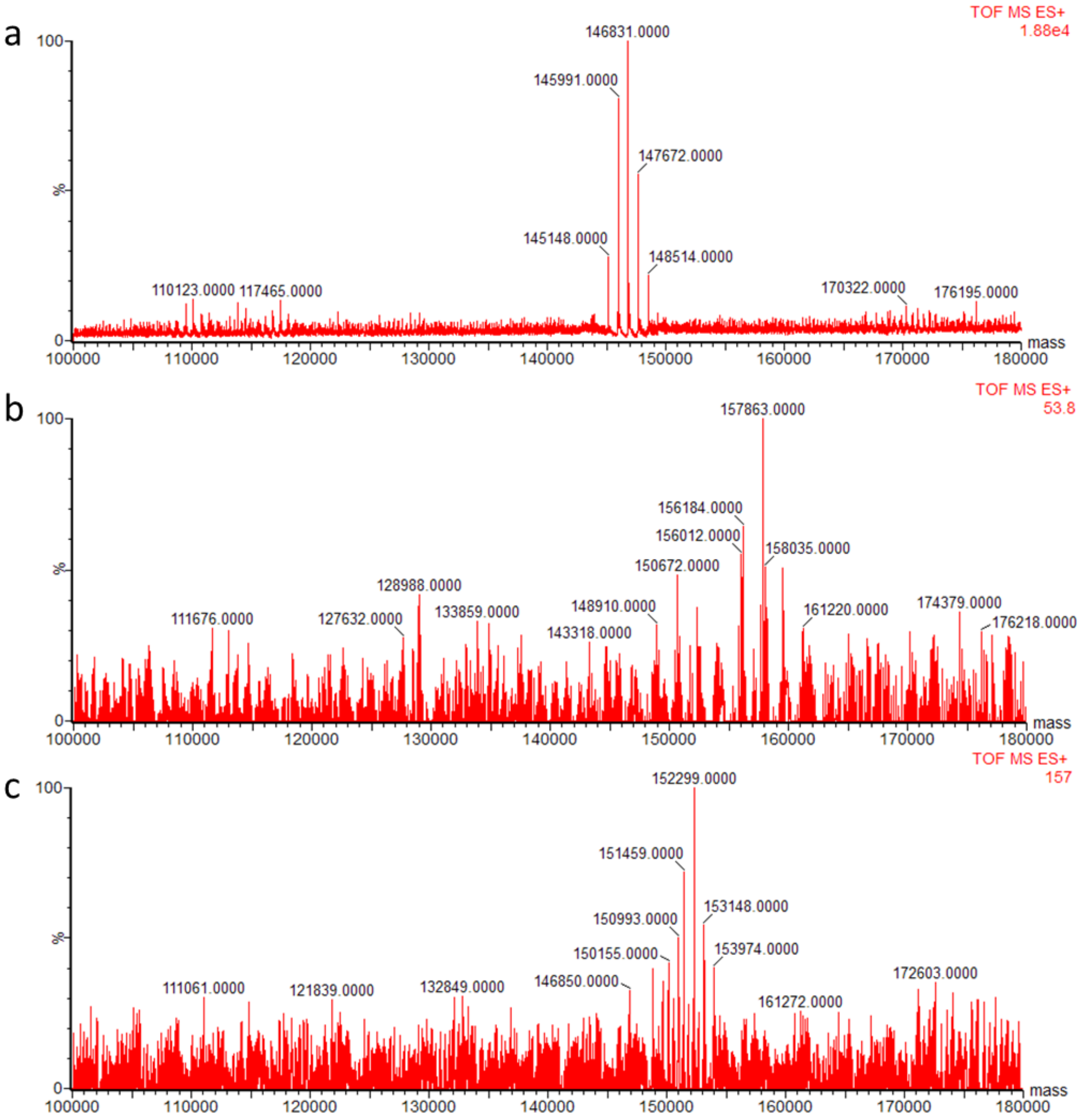

Figure S7. Mass characterization of AF647-modified Tz (**a**), Tz-Ang2 (**b**) and Tz-MiniAp4 (**c**) by LCT-Premier. Antibodies are deglycosylated with PNGase F. Deconvoluted spectra are shown.

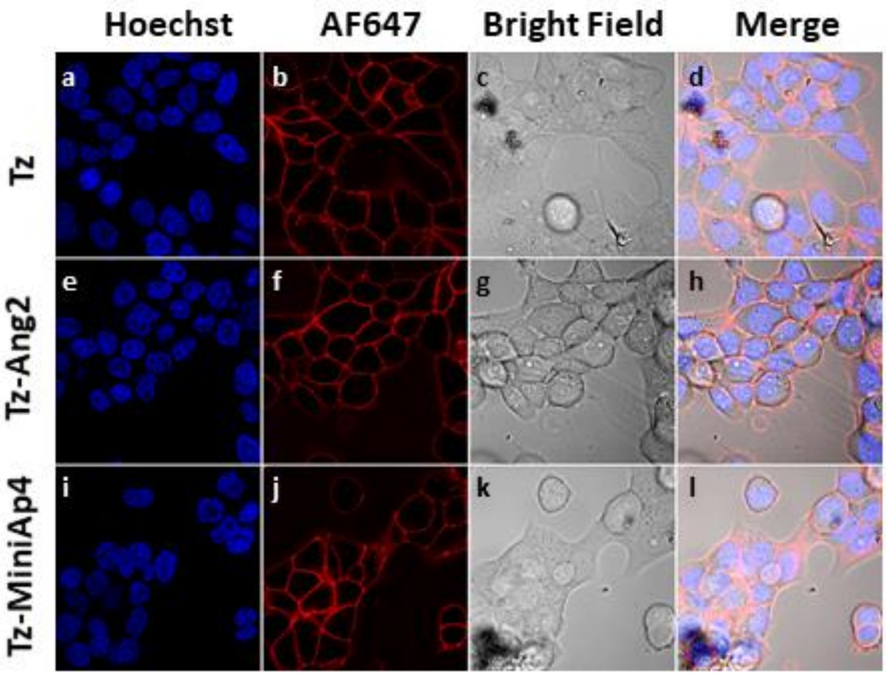

Figure S8. z-stack projection of BT474 cells incubated with AF647-Tz (**a**, **b**, **c**, **d**), AF647-Tz-Ang2 (**e**, **f**, **g**, **h**) and AF647-Tz-MiniAp4 (**i**, **j**, **k**, **l**); Hoechst nuclei staining (**a**, **e**, **i**); AF647 (**b**, **f**, **j**); Bright Field (**c**, **g**, **k**); Merged **(d**, **h**, **l**).

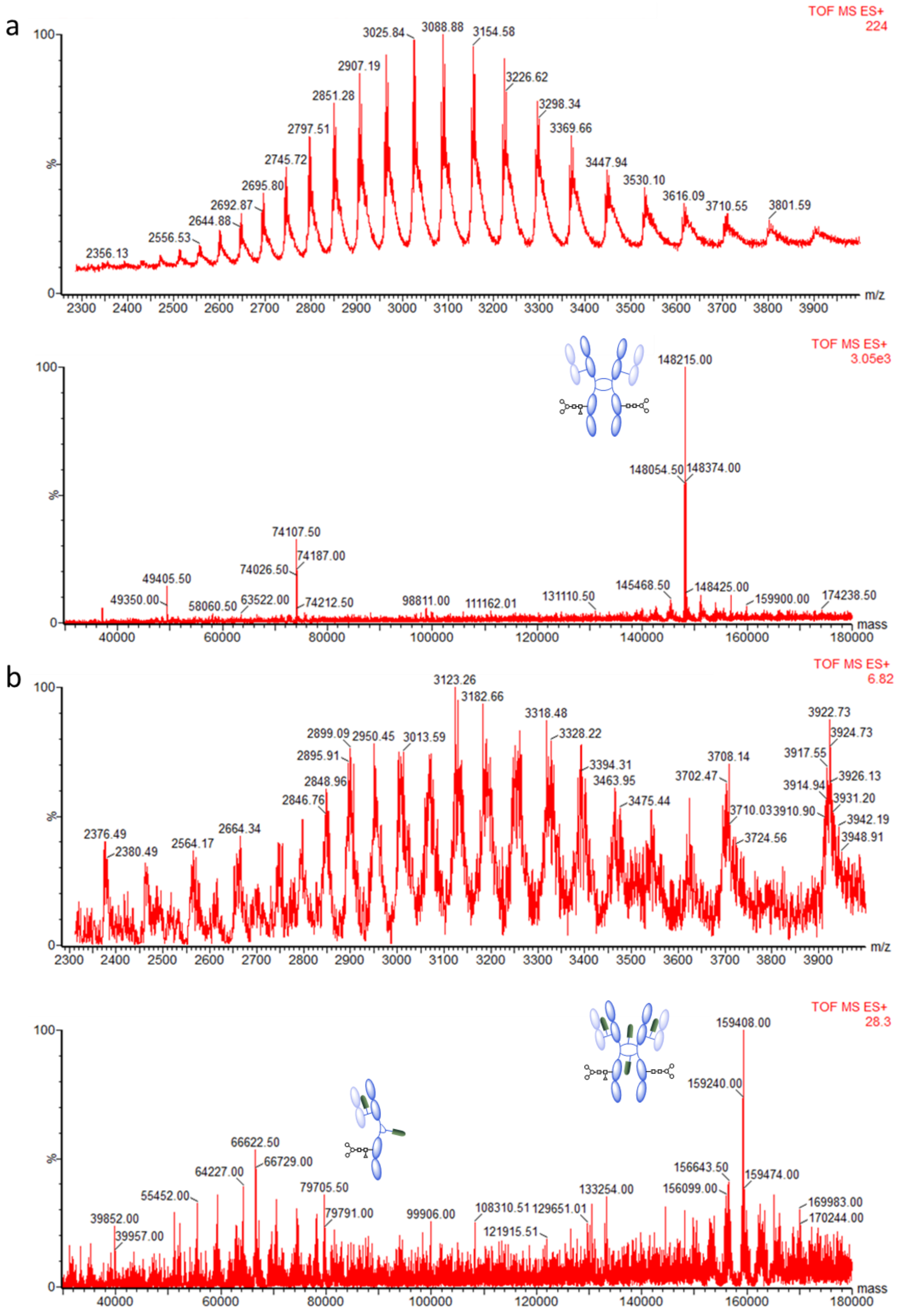
a
TOF MS ES+
224
2356.13
2556.53
2644.88
2692.87
2695.80
2745.72
2797.51
2851.28
2907.19
3025.84
3088.88
3154.58
3226.62
3298.34
3369.66
3447.94
3530.10
3616.09
3710.55
3801.59
m/z
%
TOF MS ES+
3.05e3
148215.00
148054.50
148374.00
74107.50
74187.00
74026.50
49405.50
49350.00
58060.50
63522.00
74212.50
98811.00
111162.01
131110.50
145468.50
148425.00
159900.00
174238.50
mass
b
TOF MS ES+
6.82
3123.26
3182.66
3318.48
3328.22
2899.09
2950.45
3013.59
2895.91
2848.96
2846.76
3394.31
3463.95
3475.44
3708.14
3702.47
3710.03
3910.90
3724.56
3922.73
3924.73
3917.55
3926.13
3914.94
3931.20
3942.19
3948.91
2376.49
2380.49
2564.17
2664.34
m/z
TOF MS ES+
28.3
159408.00
159240.00
156643.50
156099.00
159474.00
169983.00
170244.00
66622.50
66729.00
64227.00
55452.00
39852.00
39957.00
79705.50
79791.00
99906.00
108310.51
129651.01
121915.51
133254.00
mass

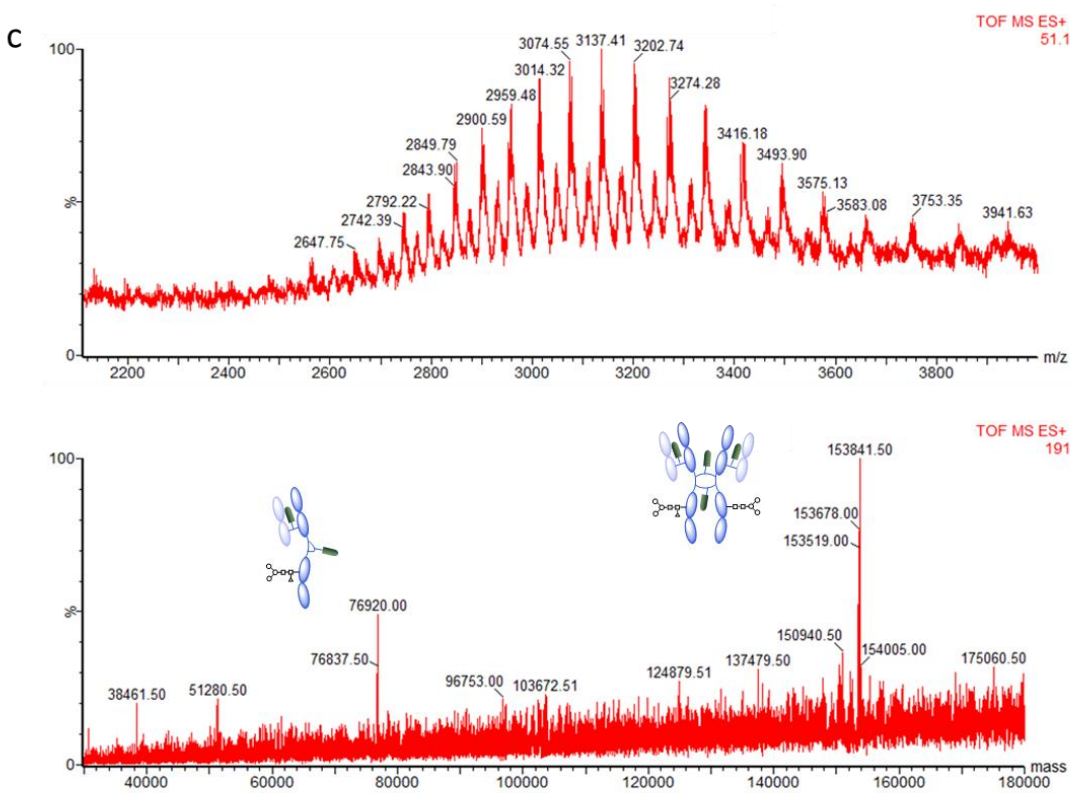

Figure S9. Mass characterization of Tz (**a**), Tz-Ang2 (**b**) and Tz-MiniAp4 (**c**) after immunoprecipitation of acceptor well from HBBBCMTA by LCT-Premier. The raw data (top) and the deconvoluted spectra (down) are shown. M_{cal} for Tz: 148212; M_{found}: 148215; M_{cal} for Tz-Ang2= 159244: M_{found}: 159408; M_{cal} for Tz-MiniAp4= 153684; M_{found}: 76920, 153678.

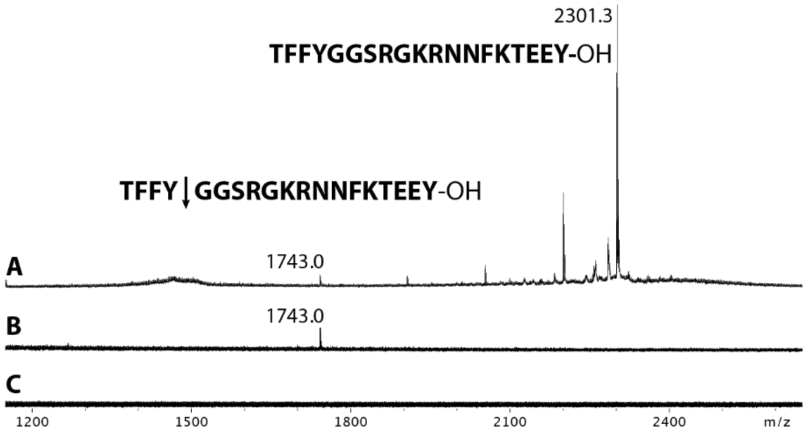

Figure S10. Degradation fragment of Angiopep-2 present in the donor (A) and acceptor (B) wells after the transport assay. A well containing only Lucifer yellow (C) as a control.

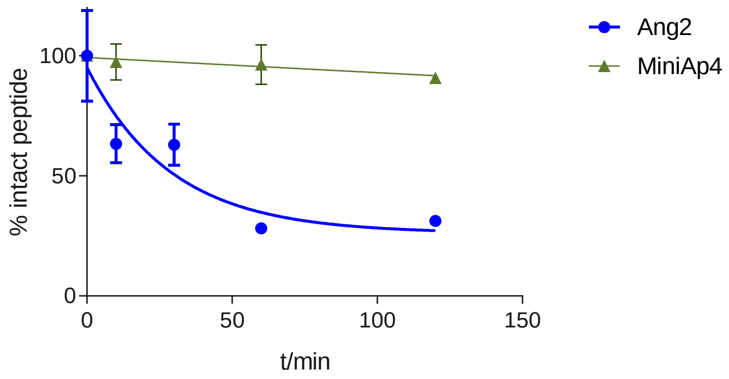

Figure S11. Stability of Ang2 and MiniAp-4 in mouse serum.

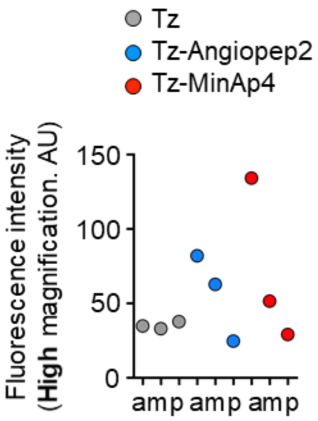

Figure S12. Quantification of NIR fluorescence from anterior (a), medium (m) and posterior (p) levels from each mouse injected with specific therapeutic antibodies.

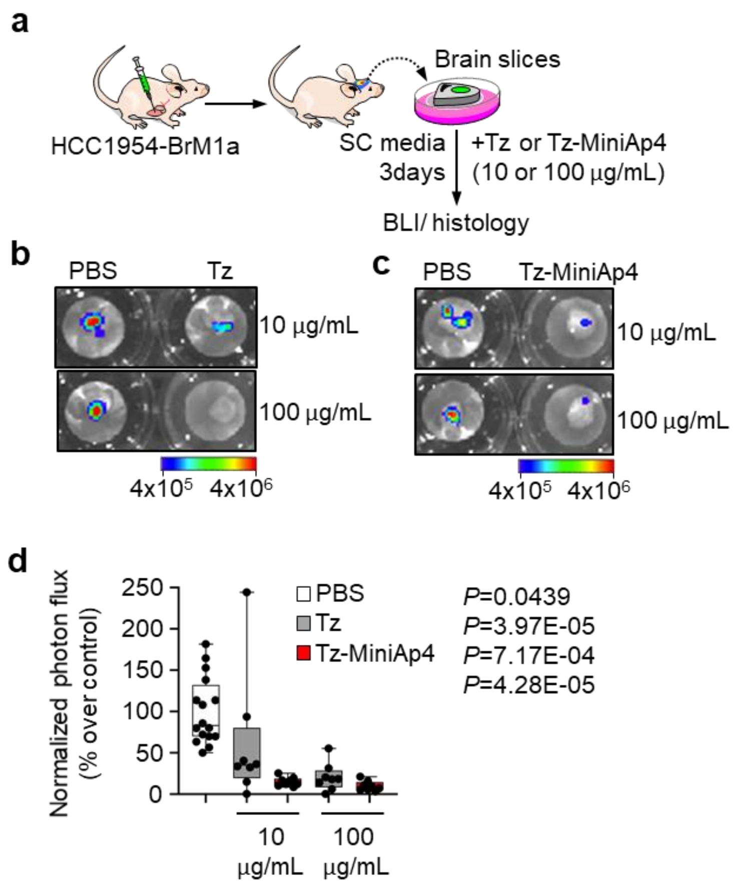

Figure S12. a) Schema of the experimental design. b-c) Representative images of brain organotypic cultures with established HCC1954-BrM1a metastases (luciferase+) grown *ex vivo* for 3 days in the presence or absence of Tz (10 or 100 µg/ml) (b) or Tz-Miniap4 (10 or 100 µg/ml) (c); d) Quantification of the bioluminescence signal emitted by HCC1954-BrM1a cells in each brain slice normalized to the initial value obtained on day 0, before the addition of any treatment. Values are shown in box-and-whisker plots, where each dot represents a different organotypic culture and the line in the box corresponds to the median. Whiskers go from the minimum to the maximum value (PBS: n= 16; Tz and Tz-Miniap4: n= 8 per experimental condition). P values were calculated using a two-tailed t-test.

Bibliography

1 J. Spengler, J.-C. Jiménez, K. Burger, E. Giralt and F. Albericio, *J. Pept. Res.*, 2005, **65**, 550–555.
